# Supplementary material for: Agent-based model projections for reducing HIV infection among MSM: Prevention and care pathways to end the HIV epidemic in Chicago, Illinois
Source: PLoS One. 2022 Oct 17;17(10):e0274288. doi: 10.1371/journal.pone.0274288 (PMC9576079; doi:10.1371/journal.pone.0274288)
Supplement: S1 Appendix — This appendix describes all details related to the implemented the LHM model based on requirement for model description set forth in the ODD protocol. (PDF) [file pone.0274288.s001.pdf]

Levers of HIV Model detailed model description

Wouter Vermeer[1,2,3], Can Gurkan[1,3], Arthur Hjorth[4], C. Hendricks Brown[1], and Uri Wilensky[2,3]

[1]Center for Prevention Implementation Methodology for Drug Abuse and HIV (Ce-PIM) at the Feinberg School of Medicine, Northwestern University, Chicago, IL, USA
[2]Northwestern Institute for Complex Systems (NICO) at the Northwestern University, Evanston, IL, USA
[3]Center for Connected Learning and Computer-Based Modeling (CCL) at Northwestern University, program in Learning Sciences and Department of Computer Science, Evanston, IL, USA
[4]Department of Management, Aarhus University, Aarhus, Denmark

Contents

1 Model Overview **3**
1.1 Purpose: . . . 3
1.2 Agents . . . 3
1.3 Variables . . . 4
1.4 Time scales . . . 7
1.5 Process overview and scheduling . . . 8

2 Design Concepts **8**
2.1 Basic principles . . . 8
2.2 Emergence . . . 9
2.3 Stochasticity . . . 10
2.4 Observation . . . 11

3 Details **12**
3.1 Model Software . . . 12
3.2 Data sources . . . 12
3.3 Initialization . . . 12
3.3.1 Data pre-processing . . . 13
3.3.2 Model setup . . . 20

4 Model behaviors and structure **24**
4.1 Networks Of Sexual Partnerships . . . 24
4.1.1 Partnership formation . . . 25

4.1.2 Partnership characteristics 27
4.1.3 Partnership dissolution 28
4.1.4 Becoming of age 28
4.1.5 Deaths 28
4.2 HIV spread dynamics 29
4.2.1 Base risk 30
4.2.2 Circumcision of the HIV-negative partner 30
4.2.3 Intra-host epidemiology, viral-load of HIV-positive partner 30
4.2.4 HIV-positive partner in the acute stage of HIV infection 31
4.2.5 CCR5 mutations of the HIV-negative partner 31
4.2.6 community level health disparities as a risk factor . . 31
4.2.7 Condom use 32
4.2.8 PrEP use by the HIV-negative partner 32
4.2.9 Calculating the per act risk of transmission 32
4.2.10 Rates of sexual activity 33
4.3 Treatment and Testing 33
4.3.1 Going in for HIV testing 34
4.3.2 PrEP assignment for those who test negatives for HIV 35
4.3.3 Retention in PrEP care for those who are HIV-negative 35
4.3.4 Re-entering in care for those with unknown status . 36
4.3.5 Testing positive for HIV while being on PrEP 36
4.3.6 Linkage to care for those who test HIV-positive . . . 37
4.3.7 Getting suppressed for HIV-positives whom are linked to care 37
4.3.8 Rates of going in for treatment for in care HIV-positives 37
4.3.9 Dropping out of care for HIV-positives 38
4.3.10 Suppression during retention in care 39
4.3.11 Becoming unsuppressed while retained in care 39
4.3.12 Re-entering into care for known-HIV-positives 40

5 Observations and model alignment 40
5.1 Validation based on incidence numbers 41
5.2 Validation based incidence distributed over age 43
5.3 Validation based on incidence distributed over race/ethnicity type 43

6 Interventions 43
6.1 PrEP linkage 44
6.2 PrEP retention 44
6.3 PrEP adherence 45
6.4 ART linkage 46
6.5 ART retention 46
6.6 ART adherence 47

This supplementary information document describes the details of model structure, processes, parameterization, and usage of data for the Levers of HIV Model (LHM). We will build on the ODD [1–3] standard of replication in documenting this model, and specifically emphasize the modular structure of this complex and high-fidelity model. This document will be structured as follows; First we present the overview of the model (section 1) and the design principles that went into it are elaborated (section 2). Next, a description of the data sources used (section 3), and an overview of all modules the details of each of these modules, and their interactions (section 4) is presented to elaborate the behavior and structure of this model. Lastly, we will discuss model alignment and validation to field data (section 5), and its use for virtual experimentation (section 6).

1 Model Overview

1.1 Purpose:

The national initiative to end the HIV epidemic (EHE) [4] has yielded various local programs that follow its twin goal of reducing new HIV infections and have all HIV-positive individuals in care by 2030. At the state level in Illinois the department of public health (IDPH) has come up with its version of the EHE plans in a program call getting to zero Illinois (GTZ) which has similarly been adopted at the city level within Chicago by the Chicago department of Public health (CDPH). And while the goals of this plan are clear, the best paths towards achieving them remains unclear, which is where our modeling effort come in.

With the existence of two highly efficacious biomedical interventions, anti-retroviral medications (ART) that greatly reduces viral load among persons who have HIV, and Pre-Exposure Prophylaxis (PrEP) that can strongly reduce susceptibility to contract HIV for those who are HIV negative but at risk for becoming infected, there is reason for optimism. However, PrEP usage is very low, and substantial numbers of people with HIV do not know they are infected, have never received care, or fall out of care. The agent-based model described in this document, which from this point onward we will refer to as the Levers of HIV Model (LHM), aims to support the decision making by local health departments in determining how to allocate their resources in achieving the getting to zero aims.

1.2 Agents

One of the main modes of transmission of HIV is unsafe sex, and in particular within the sub-population of Men-who-have-Sex-with-Men (MSM) the rates of new incidence of HIV are high. Based on CDPH surveillance

data from 2019, this sub-group is responsible for roughly 70% of the incidences in Chicago and is therefore the focus of our model. In the agents in LHM represent individual MSM in Chicago. Each agent is assigned various characteristics some of which might change over time. An overview of these characteristics is provided in section **??**. Based on demographic data, we estimate that the population of interest, MSM in Chicago, is approximately 65.000 (or 6.6% of the male population) [5]. Our model describes this population on a 1:10 scale, meaning that our model of Chicago is initiated with 6500 agents.

1.3 Variables

The characteristics of the agents in the LHM can be sub-divided into four broad categories: Demographics, HIV related attributes, treatment related attributes, and sexual preferences.

Demographics

- *Age*: All agents have an age, which is measured both in weeks and in years, which increases every time step in the model (one time step is one week). As sexual transmission of HIV is the mode of transmission studied in the model, it tracks agents over their sexually active lifespan. After initiation, the agents enter the model at age 13 (at sexual onset). Agents will leave the model as soon as they are over 80 years old or if they die.

- *Race/Ethnicity*: All agents are assigned a race/ethnicity at birth which cannot change over time. Four race/ethnicity types are distinguished: Non-Hispanic Blacks (Blacks in short), Non-Hispanic Whites (Whites in short), Hispanic, and Other. The ratios in which these race/ethnicity types occur in the model is based on demographic census data from 2016 on age and race distributions in Chicago.

- *Neighborhood of residence*: All agents are assigned a neighborhood of residence. This neighborhood is assumed not to change throughout the simulation. The neighborhood of residence is assigned based on the field data on the racial compositions of the Chicago neighborhoods in combination with the agent's race/ethnicity.

- *Being-a-tester?*: All agents have a binary variable indicating whether or not the agent will ever go in for HIV testing. Based on previous HIV modeling work [6] we estimate that 6.5% of the MSM population will never consider to go in for HIV testing. This indication of never-testing is based on a population average, and as such is assigned independent of any of the other agent attributes.

- *Circumcised?*: All agents are either circumcised or not, this variable is set at the point of entering the model, and will not change over

time. We estimated 76.8% of the MSM population in Chicago is circumcised. This indication of circumcision is based on a population average rate of circumcision among Midwest children leaving hospitals from 1979-2010 [7], and as such is assigned independent of any of race and age.

- *CCR5-mutation*: All agents have an indication for CCR5 allele mutation. A mutation in the CCR5 allele is associated with reduced risks of contracting HIV [8]. This variable can have 3 values, (0) which refers to no mutation, (1) for being heterozygous which refers to having a single mutation, and (2) for being homozygous which refers to having a double mutation. Single mutations reduce the risk contracting HIV, whereas double mutations render the agent completely resistant. Rates of mutations vary across race/ethnicity with Whites more often being affected by this mutation [6].

HIV Attributes

- *HIV status*: All agents have an HIV status. They can either be HIV-positive or HIV-negative. Note that once positive an agent can never go back to being HIV-negative. The HIV-status indicator is independent of whether that status is known.

- *Viral-load*: All agents have an indicator of their viral-load, which describes the number of copies HIV that are in 1 ml of their blood. By default HIV-negative agents will have zero copies.

- *HIV in acute stage?*: All agents have a binary indicator of their HIV infection being in the acute stage or not. HIV infections progress in 4 stages (more on this in sections 4.2.3 and 4.2.4), of which the first two are considered acute. In the acute stage the there is an excess risk of HIV transmission above that predicted by the heightened viral-load during that period.

Treatment attributes

- *HIV+ status Diagnosed?*: All agents have a binary variable indicating if their HIV-positive status has been diagnosed. By default this value is FALSE for all HIV-negative individuals. Once an HIV-positive agent goes in for HIV testing, and it found to be positive this indicator is switched to TRUE, and the agent is considered a known HIV case and be part of the people living with HIV (PLWH).

- *linked-to-care?*: All agents have a binary variable that indicates if they are currently linked to care. To qualify as linked-to-care that agent needs to have had a HIV test, and have entered the treatment cycle. For HIV-negatives this is equivalent to being exposed to and potentially get on Pre-exposure prophylactics (PrEP). For HIV-positives

this is equivalent to getting a CD4 viral load test, after their initial positive diagnosis.

- *In-Treatment?*: All agents have a binary variable that indicates if they are currently in treatment. Once linked to care, an agent is considered to be in treatment (regardless of the effectiveness of such treatment). An agent remains in treatment until he fails to be retained.

- *On-PrEP?*: All agents have a binary variable indicating whether they are currently on PrEP.

- *weeks left on-PrEP*: As PrEP is prescribed for a limited duration, each agent will have a variable indicating for how long (in weeks) their PrEP prescription is still active. In our model the default prescription duration for PrEP is 13 weeks. When the prescription is not renewed prior to these 13 weeks passing, the individual is considered to have dropped out of care, and to no longer be on PrEP (their on-prep? variable will be set to FALSE).

- *Adherence level for PrEP*: Each agent will be assigned a level of PrEP adherence upon birth, this level will not change over time. This categorical indicator specifies the extend to which the agent will adhere to PrEP medication if he gets on PrEP. PrEP is currently provided in the form of a daily pill, and not all individuals will be able to stick to this regiment. We identify four levels of adherence; non-adherent (take non of the pills per week), poor adhering (taking less than 2 pills per week), moderate adherence (between 2 and 3 pills per week), and full adherence (more than 4 pills/week). Each of these levels of adherence is associated with a certain level of protection against contracting HIV [6].

- *HIV+ Suppressed?*: All agents have a binary variable indicating whether or not their viral load is suppressed or not, suppressed viral loads are associated with reduced risk of infection. Upon being tested positive and being in care, agents will put on antiretroviral treatment (ART). When effective in adhering to the treatment their viral load will become suppressed, and the suppressed variable will be set to TRUE for as long as the agent remains retained in care.

- *weeks suppressed*: Once an agent becomes suppressed it takes some time before viral loads are reduced to suppressed suppression levels. As such each individuals has a variable indicating for how long they have been suppressed. This parameter is used to calculate the appropriate viral load levels.

- *Retained-in-care?*: Once in care, each agent will have a rate at which they will have a care visits. For as long as the agent indeed goes in for these care visits he is considered retained in care. However, ever

so often agents will make the decision to not go in for their care visit which would result in that agent no longer being retained in care.To keep track of this status each agent will have a variable specifying if he is currently retained in care or not.

Sexual preferences

- *Sex-role*: Each agent is assigned one of three sex-roles at birth. An agent is either Insertive, meaning he will always and only engage in insertive sex acts; Receptive, meaning he will always and only engage in receptive sex acts; or Versatile, meaning he can engage in both insertive and receptive sex acts. The sex-role of agents does not change over time.

- *Insertivity-preference*: All agents have an insertivity preference, which indicates how likely they are to engage in insertive sex acts. This preference is 0 for receptive agents, and 1 for insertive agents. Versatile agents will have insertivity value between 0 and 1.

- *Sexual activity*: All agents have an indicator of their sexual activity. This is a categorical variable that indicates the quintile of activity of the agent; e.g. being the top 20% most active agents with the given age/race/HIV-status/sex-role. As we consider it possible that agents become more or less active over the course of time each time an agent ages a year, there is a 2.0% chance to either move to an adjacent quintile and become more or less promiscuous.

1.4 Time scales

The LHM progresses trough time by means of discrete steps, or ticks. It adopts behaviors at four different times-scales, of which the primary one, the tick, represents a week in the real world. The second time-scale that is used in the model is one that represent days, as there are some daily behaviors in which agents engage. Specifically, individual level decisions regarding going in for testing are made on a daily basis and consequently aggregated to the week level into a decision to do a test in a given time step. The third timescale used in this model is the yearly behaviors of the model. We validate the behavior of the model (in terms of new HIV incidence) based on yearly trends, and hence consolidate the model behaviors over 52 time steps (a year) to align our model to field data. Lastly, the fourth timescale we use is for making long term predictions about system-wide behaviors; here we consider the behavior of the model over a period of 15 years (780 time steps), representing the years 2016 to 2030 to look at trends in longitudinal impact of policy interventions. The LHM does not include any spatial dimensions.

1.5 Process overview and scheduling

In this section, to provide an overview of the steps in the LHM, we provide a high level pseudo code of the behaviors. First the model is setup, after which the dynamics of the model progress in discrete time steps (ticks). Within a tick, multiple behaviors occur. All agents complete a single behavior before the next behavior is started, and for each behavior agents are activated in random order. Note that the description below is only meant to provide an overview, a more detailed description of each the actual behaviors in each of these steps is provided in the sections 3 and 4 below.
In each tick:

1. Agents get older and potentially die
2. Agents go in for treatment and potentially drop out of treatment
3. Agents go in for HIV-testing and potentially get linked to care
4. Sero-discordant couples —couples in which one partner is HIV-positive and the other is HIV-negative— determine their sexual activity
5. Sero-discordant couples potentially transmit HIV during their sex acts
6. Agents break the relationships that have run their course
7. Agents start new relationships
8. Agents start new one-night stands
9. New agents enter the model

2 Design Concepts

2.1 Basic principles

This model is based on the fundamental notion underlying all epidemic models; interactions among individuals can cause viral spread within a population. It considers one specific mode of transmission of HIV, transmission by means of unprotected anal intercourse among MSM. Traditionally such a process has been modeled using mean field compartmental mixing models which assume rates of interaction to be homogeneous within populations, an assumption that does not align with the observation that networks of interactions among agents can be strongly heterogeneous. In contrast to such a mean field approach, our model considers an agent-based approach, capturing the heterogeneous dynamic sexual behaviors among MSM at the individual level, and letting a network structure emerge from them. the resulting emergent network structure evolves dynamically based on individual level partnering decisions made at the agent-level. Agents will make partnering decisions based on their own individual preferences and attributes

resulting in two types of interactions; Long-term ties, and one-time ties. The latter are ties that only persist for one week, whereas the prior are ties with a length beyond that persist over multiple ticks.

Based on the individual level partnering decision a network of sexual interactions will emerge. On top of this sexual network we assume a model in which sero-discordant pairs (partnerships in which one partner is HIV-positive and the other HIV-negative) have sex and each sex act can cause the HIV virus to spread. Within a sero-discordant pair there are various biomedical and behavioral factors impacting the per act risk of transmission of HIV. Details on these risk factors will be provided in section 4.2 below.

We aim for the LHM to primarily serve as source for decision support for local health departments. It can be used to provide predictions on the trends of the HIV epidemic and care cascade under various modeled interventions in the care continuum. Such computational experiments provide a quick and inexpensive way to gain insights to supporting decision making. By identifying the most efficient levers to change the model results help steer the epidemic towards the intended targets, and provide input for selection of the appropriate intervention portfolio and strategies to achieve local HIV prevention and reduction goals.

2.2 Emergence

As briefly touched upon, all the behaviors in this model are based on individual level decision processes. As such, all system level dynamics can be considered to emerge from simple agent level rules. In particular the network structure, and all the elements depending on that structure, such as incidence (the primary outcomes of the model), and the state of the world at a given point in time should be considered emergent.

The network structure is the result of partnering decisions made by individuals. At each time step, each individual determines if and how many new relationships it wants to form of each type. Then all agents that want to create a given type of tie are pooled and they will try to find a match in that pool of "tie seekers". In random order these tie seekers will be activated to find a partner from the pool. When looking for a partner the agent (ego) will have a preference for a given partner based on a set of characteristics of their ideal partner, and will score all potential partners (alters) based on how well they match to the ego's ideal set of attributes. Similarly all alters (who have their own preferences) will score the ego based on their preferences. Based on the dyadic match (the product of these matching scores) the likelihood of the ego selecting the alter is determined, after which the ego does a weighted random pull based on these likelihoods. This process is repeated until the pool of tie seekers is empty. As a consequence the network structure truly

emerges from the local behaviors. The new incidences of HIV in the model are the result of the emerging network, the characteristics of the individual agents (the world state) which in itself is path dependent and emerges, and the stochastic inter-host spreading dynamic process on top of the network.

2.3 Stochasticity

As touched upon in the previous section, many of the processes in this model have an element of stochasticity in them. Stochasticity first comes into play in the initiation of the model. We use a demographic file which describes every individual in Chicago and has their Race/Ethnicity, gender, age, MSM status and HIV status. As the model describes the city on a roughly 1:10 scale, upon initialization of the model a subset of these individuals is randomly chosen to be included in the model. During the initialization, the following set of characteristics is set in a probabilistic manner: Neighborhood of residence, being a tester, circumcision status, CCR5 mutation, Viral progression (if HIV+), in-care status (including PrEP use and Suppression), PrEP adherence level, Sex role and sexual activity quintile. As these attributes are assigned in probabilistic fashion, this implies that the world state is always the result of a stochastic process and none of the mean characteristics are guaranteed in a single setup.

Other areas that are explicitly stochastic (in the sense that the rate at which they occur varies) are; the rate of partnership formation and dissolution, the dyadic behaviors, risk of transmission, and treatment effects. We briefly elaborate each of them below, and will provide more details in sections 3 and 4.

- **Partnership formation and dissolution**: Each individual has a given rate at which it will try to form new ties, which is conditional on the activity quintile, race/ethnicity, age, and HIV status. They will consequently determine the actual number of new ties being formed that tick from by sampling from a Poisson distribution with a mean based on that rate. What is more, once the tie is formed, it will be assigned a duration based on a distribution of tie durations, also making the persistence of ties a stochastic process. With both formation and dissolution being stochastic, and partner selection being a weighted random pull based on matching scores, the overall structure of the network can be considered stochastic.
- **rate of dyadic behaviors**: Similar to the duration of the tie, the rate of various attributes within that tie are determined stochasticly upon creation of a partnership. The rate of sexual activity, insertivity, and condom use are all preset for that tie. Consequently these rates are

then used as means of Poisson distributions which are consequently sampled each tick to obtain the actual behavior.

- **Risk of transmission**: For each sex act the actual behaviors are modeled in order to come up with a risk of transmission. This risk of transmission is consequently used as the success chance in a single Bernoulli trail, making transmission in each sex act a stochastic event, and consequently the actual HIV spread a stochastic process.

- **Treatment effects**: All mechanisms related to treatment and prevention (ART and PrEP respectively) are based on CDPH surveillance data and the RADAR cohort study [9, 10] respectively. This data describes the behaviors of our population stratified by age and race/ethnicity type. Based on these population level characteristics we devise the rate at which either treatment or prevention events will occur, which are consequently translated to stochastic processes on the individual level per tick. The processes that are specifically stochastic within the context of treatment are; going in for HIV tests, going in for treatment, being linked to care (both for HIV+ and HIV-), being retained in care (both for HIV+ and HIV-), getting on PrEP, and becoming suppressed.

2.4 Observation

The LHM is intended to provide support to local health departments' decision making with regards to making their Getting to Zero (GTZ) aims. These aims primarily relate to obtaining rates of new incidence that are near zero. For Chicago specifically this is considered to be at 10% of the baseline rate of 2018 when GTZ plans were initiated, effectively meaning fewer than 56 cases by 2030. In line with this aim, the primary outcome observed from this model is the number of new incidences annually. More specifically, as the measure that CDPH captures only those incidences which are *observed*, we measure the observed incidence, which will in fact differ from actual incidence. We will make corrections to the incidences numbers obtained from our model to align them, in meaning, to the incidence numbers captured by the CDPH.

Beyond the total number of incidence cases, we are interested in the distribution of those incidence cases. As such we output the distribution of the incidence cases both across different ages and different race/ethnicity groups. For all three outcomes we have CDPH counterfactual information (for the year 2016), which is used to validate the first year of behavior in our model on the system level. The following 15 years of modeled behavior are consequently used to predict trends and impacts of intervention strategies.

3 Details

3.1 Model Software

The LHM is built in NetLogo [11], a multi-agent programming language and modeling environment for simulating complex natural and social phenomena, it is particularly well suited for modeling complex systems evolving over time. The model is implemented in version 6.2.2 of NetLogo. The NetLogo model uses various input files to process the field data, these input files are generated using various scripts in R (Version 3.5.1) and these files are included in the model code.

3.2 Data sources

The LHM, being a high-fidelity model, relies heavily on field data to align the behaviors of its agents to those observed in the real world. To do so, various sources of data have been used; the four that cover the majority of the dimensions are:

1. Demographic data of the city of Chicago based on census data for 2016: This data is the main source used to devise a population of representative agents in our model

2. RADAR cohorts study data: RADAR is a Chicago based longitudinal cohort study [9, 10], it contains sexual network data spanning 3 years and covering more than 1,200 MSM. This data is used to provide information on sexual partner formation processes among MSM in Chicago.

3. HIV care cascade data from CDPH: This data describes the extent to which treatment is provided to the population of interest. This data is used as the main source to inform the treatment provision modules and attributes

4. HIV epidemic data from CDPH (summarized in [12] and preceding reports): This data describes the HIV epidemic on the city level and is used to validate our models' behavior against.

3.3 Initialization

The initiation of the model for experimentation can be divided into three different and relatively independent stages. In the first stage, which we will refer to as the data pre-processing stage, we will take the available field data and convert these to input data required for our model. In the second stage, which we will refer to as the model setup, we will use this input data to generate a starting instance of the world, e.g. create agents and a network of interactions. In the third stage, which we call the burn-in, we make sure the model behavior has stabilized prior to starting any experiments.

3.3.1 Data pre-processing

To initialize the LHM we pre-processed the data from three particular sources: First, we used the demographic data to prepare a list of all individuals in the City of Chicago, with characteristics that is representative for the demographics observed. Second, we used the information from the RADAR study to inform various aspects of the network formation and dissolution process. And Third, we used CDPH data to create a local risk inflation factor, conditional on the neighborhood of residence. We will elaborate on each of these steps below.

Generating a population file

The population file describes all individuals in Chicago, categorized by basic demographics (race-ethnicity, sex, and age), MSM status, HIV status and mode of infection. Unless explicitly stated, all the data below were generated using data from Chicago or Cook County census.

Basic Demographic Information: We retrieved race-ethnicity, sex, age (by year starting at birth) denominators from the City of Chicago for 2016. We produced a dataset with each record representing a live person in Chicago. Race-ethnicity was categorized into HISPANIC, NON-HISPANIC BLACK, NON-HISPANIC WHITE, and OTHER. The total number of individuals in Chicago was 2,655,422. To account for aging of individuals for future years, we produced 15 years of projected race-ethnicity by sex births using the same distribution and numbers for live births in 2016. These additional individuals could be used for long-term projections in the model if needed.

MSM Status: MSM status is added to individuals as they reach age 15. The proportion of males who were MSM was taken as 0.066, independent of race-ethnicity. This proportion was taken from [5], for Cook County (which includes Chicago), based on data from the American Community Survey.

HIV Status: We coded individuals based into the following categories, Known HIV Positive, Not Known HIV Positive, and HIV Negative. The total of known or unknown HIV Positive in Chicago between ages of 13 and 69 was estimated as 25,140 as of June 2018. We obtained from the Chicago Department of Public Health the number of known HIV prevalent cases in Chicago by race-ethnicity, age grouping, and mode of infection. As our current calculations involved only MSM, we combined sexual transmission of MSM and injecting drug use among MSM). Numbers were distributed equally across all ages within each age category. We then computed probabilities of known HIV by race-ethnicity and age for MSMs in Chicago using Bayes Theorem, the prevalent cases and the denominators. Individuals were then assigned as known HIV according to these proportions.

To account for unknown HIV positive status, we were not able to project these numbers based on Chicago data alone because only marginal totals were available (e.g., sex, race, transmission category). Instead, we relied on estimates of the proportion of known HIV to total HIV by age, which showed a strong relationship. For example, 55.6% of 17 year-olds with HIV were estimated as knowing that they were HIV, whereas 93.7% of those aged 70 knew they were sero-positive. We used data from these 6 age-related proportions and transformed both the response and age to form a linear relationship. In particular, we fit:
$Y = \frac{PNKP}{PKP} = (\frac{1}{PKPOP} - 1)$ to $\log(Age - 10)$ using a linear model with intercept = 1.47697 and slope = -0.36198. This produced an R^2 of 0.97. In which PNKP is the Proportion Not Known HIV Positive, PKP is the Proportion Known HIV Positive, and PKPOP is the proportion of known HIV positive to all HIV positive.

Network formation and dissolution mechanics from RADAR

The RADAR cohort study, in which a Chicago based cohort of 1200+ MSM were recruited to every 6 months to come in, get HIV tested, provide information about their sexual risk behaviors, and partnership network. While the RADAR project has generated a wide range of data, we specifically use three files to inform our model:

1. An edge list containing all the sexual ties formed during each for the first 6 waves of data in the project. Each edge contains a set of relationship attributes, alter attributes and an Ego ID. We will refer to this type of data as the "relationship data".
2. A list of individuals that reported to have no sexual ties during the RADAR interviews. In network terms these are the isolates per wave of data. We will refer to this data as the "isolates data".
3. A file containing the individual level characteristics of each individual in the data set for each wave of data. It describe for each agent's visit, their attributes, and various questionnaire answers, of which we only use a limited set of items. We will refer to this data as the "ego data".

Consolidating the relationship records

The first step was to consolidate the edge list of relationship data. RADAR data is collected in waves that each roughly represent an observation period of 6 months. Consequently, relationships that span a period longer than 6 months will be represented as multiple records within the relationship data. Without pre-processing this would result in an overestimation of the number of relationships that individuals have and underestimation of the duration of their relationship. To cope with this, multiple records of a single relationship were consolidated into a single record. We, for each unique pair of ego and alter, filtered all records, and for each record in the filtered set we:

- For each record we convert the duration from days to weeks. To distinguish between one-night-stand and relationships, we take the base rate of days, reduce it by 1 and divided it by 7 to obtain the number of weeks, and take the smallest integer not smaller than our calculated value. E.g. 0 up to 1 days will convert into 0 weeks, 2 up to 8 days will convert into 1 week, and 9- 15 days will convert into 2 weeks and so on.

- For each pair of agents we determine if there are duplicate records in the data-set. A duplicate occurs when the pair has a relationship in multiple waves of data, hence duplicated segments indicate that the relationship existed for a period longer than 6 months.

- For each record that has duplicates, we create a new compound record to capture this relationships.

- For all compound records, we assign the sum of the durations of the set of duplicate segments as the duration of the compound record.

- For all duplicate records that have a duplication record in both the wave prior and following (and are thus in the middle of middle of the relationship) we add a full duration of 26 weeks (6 months) to the compound record.

- For all compound records, we assign the sum the anal sex acts of the set of duplicated segments as the number of anal sex acts in the compound record.

- For all compound records, we assign the sum of the unprotected anal sex acts of the set of duplicated segments as the number of the unprotected anal sex acts of the compound record.

- For all compound records, we determine if the relationship duration can be exactly determined or needs to be estimated. If first duplicate relationship segments indicated the relationship was preexisting, or when the last segment has specifies no end of the relationship, we have no way to determine the exact duration of this relationship. We indicate these cases as needing an estimated duration.

- For all compound records, we then replace the duplicate segments, by the newly created compound record.

Estimating the relationship durations

After compounding we have set of unique relationships. For some of these relationships the duration needs to be estimated. While these relationships do have a lower bound duration as for each segment compounded their duration will have increased by 26 weeks. For each of these records we estimated

the duration in the following manner:

For each record in each group of durations, starting with those with lowest lower bound (at least 6 months), we took the existing distribution of known durations (those for which we know the exact duration), and fit a gamma distribution to this data. For each record of unknown duration we then randomly sample this gamma distribution until we obtained a value that was higher than the lower bound and replace the duration of the compound record with this new value, we then consider the duration of this record as known. This effectively provides the right tail of the distribution based on the known left side of the distribution. We record the factor by which the original duration is inflated during this step and use this factor to inflate the sexual activity (number of anal sex acts and number of unprotected anal sex acts) accordingly. After the first group (those with a lower bound of 6 months) we repeated this process records with lower bounds of 12, 18, 24 and 30 months respectively, until all durations are known, and relationship data has been consolidated.

Merging Ego and relationship data
Next we linked the relationship data with the ego data as the relationship data describes Egos only by their ID (not their characteristics). To have all information regarding Egos in the same data frame this information needed to be merged with Ego data file, which was consequently done by matching on the Ego ID and the visit number. We took the Ego ID and visit number, looked up the record in the Ego file with matching values, and appended all information from the Ego file to the corresponding relationship record. If a record could not be found we appended the same attributes and replace their values by NA, such records were excluded from analysis.

Distilling the network attributes needed for the HIV model
Next we focused on the network attributes used in the LHM. To get the agent-based model functioning properly a static snapshot of the network is insufficient, instead we needed to accurately map the dynamics of the network of sexual interactions. The first component of such dynamics is the rate at which new relationships are being formed.

We considered the rate of new tie formation to vary based on age, race/ethnicity , HIV-status, and other individual characteristics that would be captured in an activity quintile. For each of the of these cross tabulated categories we estimate and record the rates of tie formation and use this as an input file for our model. The rate of tie formation for each category (a combination of attributes age/race/HIV-status/activity quintile) is based on the observed number of ties in the relationship data.

- For each combination of race/ethnicity, age, HIV-status we determine

how many individuals were present in the RADAR data. For each of these individuals we determined the number of ties created. And record this number in a vector of activities for this scenario. In determining these counts we distinguished between the formation of two types of ties; one night stands (those that last up to one week) and relationships (those that last longer than a week)).

- We use the isolates data include records matching the scenario as observation of no tie formation. Similar to the relationship data, in the isolates data, we filter the records in which the ego characteristics matched the scenario. We count how many unique egos are present, and append a zero for each of them to the formation activities vector of this scenario.

- The resulting vector of formation activity (per 6 months) for each scenario, was split into 5 levels (quintiles) by taking the middle of each quintile (10, 30, 50, 70, 90 quintiles respectively) of activity. Theses means are consequently used as representing the activity of that quintile. To ensure we have a distribution of quintiles to estimate we add the requirement that at least 2 observations of activity need to be present in each scenario (if this criteria is not met we considered data of this specific scenario to be missing data).

Extrapolating the rate of tie formations from field data
The previous steps resulted in a distribution of sexual activity among the scenarios present in the RADAR data, yet LHM requires input beyond this as it covers scenarios not present in RADAR data (specifically for older age groups). RADAR covers individuals that roughly span the age range 16 to 30 (very limited data for ages 30+ is present). This means that the age range of the data does not match the age range in our model; the model will cover MSM that will age well beyond 30 years old. For these age ranges (and some other specific scenarios with missing data) tie formation rates are missing and consequently need to be estimated based on the data we do have.

In exploring how to best extrapolate this missing relationship formation data, we found the rate of new tie formation to be relatively stable across ages. E.g. for a given scenario (and ego that is non-hispanic black, HIV+, and in the 5th quintile) the rate of forming new ties seemed to only vary slightly across the observed ages (we did notice drop offs in the tails of the age ranges, indicating younger and older MSM seemed slightly less sexually active). This observation was then leveraged to populate data for scenarios with missing data. To populate these scenarios we first anchored the rates of tie formation to 0 at ages 13 and 75. Meaning that by definition all agents younger than 14 years old and older than 75 years old will not form any new ties. Next we looped over the following steps until all scenarios had an associated tie formation rate:

1. We determine which scenarios with missing data and stored this list.
2. We randomly pick one of the scenarios from that list.
3. We find all observations of tie creation frequencies (in the current relation formation rates data) that match this scenario in terms of race/ethnicity, HIV-status, and activity quintile, and that are within an age-range of 10 years of the selected scenario.
4. We take a rounded weighted average from these rates, and consider that to be the rate for the selected unknown scenario. The weight here is set to 1 divided by the absolute distance in age of the observation relative to the selected scenario. This makes sure that ages close to the selected scenario have a relatively strong impact, and that rates of similar ages do not vary too strongly. Due to the anchoring at 0 in the extreme ages this results in a smooth drop towards 0 in the anchors.

Once completed this process yielded two output files, which were consequently used as inputs for the agent-based model. One file with tie formation rates for one-night stands and one for relationships, in which for each scenario (a combination of age/race/HIV-status/activity quintile) a rate of tie formation is specified.

Extrapolation of relationship data for ages beyond those found in the RADAR data

Similar to the rate of tie formation we ran into a problem with the age range in the relationship data. RADAR covers only ties of egos that range up to age 30, with some very sparse data for ages 31-32. As our model aims to cover MSM that go well beyond those age ranges, we needed to extrapolate the existing data and generate records our model can sample from for those ranges.

To do so we again generated a list of scenarios based on ego's age, race/ethnicity, HIV-status, and sex-role. To identify the ego's sex-role in the existing relationship data, we first calculated the proportion of insertive sex acts within each record in the relationship file. A proportion equal to 1 refers to this tie being purely insertive, proportion 0 indicates a purely receptive tie, and any value between 0 and 1 refers to versatile ties. For all ego scenarios with age ranges 13 - 80 (the range for which we aim to generate simulated data) we took the following steps and repeated them 50 times, Generating 50 observations for each scenario (this number is independent of how likely such a scenario is to occur):

1. For each scenario, we estimated the expected age of alters:
    (a) For each age represented in the RADAR data (16-32) we selected all records that match in terms of race/ethnicity with the ego scenario being estimated.

(b) For each of these records we stored all alter ages. This yielded an alter age distribution stratified by race and age; e.g. we know the alter ages of all ties of individuals that were Black and 18 years of age.

(c) Exploration from these alter age distributions showed a near linear progression of alter age as egos age increases. Consequently these distributions were used to estimate a linear model of alter age as a function of ego's age stratified by ego's race/ethnicity.

(d) This linear model was then used to estimate an alter age for each simulated record.

2. For each race/ethnicity we estimate race/ethnicity homophily. For each race/ethnicity we selected all records in the RADAR data that matched race with the ego scenario being estimated. For this set of records we stored the alter races in a list. E.g. a vector of the race/ethnicity types of all alters of White egos. As this is the population of all alter race/ethnicity types, we randomly sample from this vector to obtain the alter race/ethnicity of the simulated records. In doing so the racial mixing and homophily observed in the field data is maintained in the simulated records.

3. With both an alter age and race/ethnicity determined for the simulated records, we sampled the other tie characteristics for each simulated record. To do so we found all records in the RADAR data that match the simulated record in terms of ego race/ethnicity, HIV-status, and sex-role (note that age is the dimension was used to extrapolate over, and hence it was ignored in this selection process). We then randomly sampled one record out of these data, and use it as a template, copying the number of anal sex acts, the proportion of insertive sex acts, the likelihood of condom use, the duration of the relationship and the HIV status of the alter, from the existing record in the RADAR data to the simulated record.

4. We append the simulated relationship data to the original RADAR relationship data to create one single file that covers the full range of potential partnerships for all ages, race/ethnicity types, sex-roles, and HIV-statuses.

This relationship file is the fourth file that we use as input for the LHM.

Generating a locally based health disparities module

The LHM like any model is an abstract representation, in this case of the sexually based HIV transmission dynamics among MSM in the city of Chicago. While the model tries to be as accurate as possible there are certain factors that cannot be captured by the individual level behavior. There are various social factors that are not directly captured in the model, ones that particularly stand out are the racial segregation in Chicago, the disparity

in socio-economic opportunities, and access to healthcare that go hand-in-hand with it. As a result of such location based factors spatially distributed health disparities that are critical to consider for local policy might be left out when poorly capturing these dynamics. For this reason the LHM incorporates a module that captures potential health disparities that locally based.

This effect is modeled by adding a factor to the calculations for risk of transmission during each sex act which represents the relative local risks, or risk inflation factor. This factor is serves as a proxy for locally based health disparities, and varies by neighborhood of residence. Effectively depending on an agent's neighborhood of residence it will increases/decreases the per act risk of contracting HIV.

The numerical coefficients of the risk inflation factor is based on combination of three factors, the product of a proxy of the neighborhood level viral load, defined as the product of community HIV prevalence multiplied by community rate of non-suppression, and a proxy of socio-economic factors captured through the community hardship index, and a fitted normalizing factor to ensure the systemic levels incidence are maintained. While this implementation captures the major regional health disparities observed, it largely obscures what drives these disparities as this is out of scope for the current version of the model. The resulting coefficients, stored and used as a fifth input file for LHM, are consequently used as an risk inflation factors impacting the probability of contracting HIV.

3.3.2 Model setup

Setting up the model starts with reading in all external data described above. During pre-processing stage five input files were created.

- A one-night-stand frequency file containing rates of new one-night-stands created by agents stratified by Age, Race/ethnicity, Sex-role, HIV-status, and sexual activity quintile.
- A relation formation frequency file containing rates of new relationships created by agents stratified by Age, Race/ethnicity, Sex-role, HIV-status, and sexual activity quintile.
- A link attribute file with the attributes of potential links formed by agents stratified by Age/Race/Sex-role/HIV-status, containing both one-night-stands and relationships.
- An individuals file containing the complete list of people in Chicago, with their associated attributes: Age, Race/ethnicity, gender, MSM status, and HIV-status.
- A local risk inflation file that contains the factors by which the risks of contracting HIV are inflated depending on neighborhood of residence.

Although setup does not require the complete files to be read in, all data during setup is stored withing the model. While this increases the memory use of the model it speeds up processing time, which makes it more efficient for longer runs. On top of these pre-processed three additional data files are read during setup.

1. The first provides the mortality rates for the population, stratified by race/ethnicity type, age, for the general population, based on cook county mortality rates
2. The second provides the same information specifically for HIV positive individuals based on CDPH data on the mortality of People Living With HIV (PLWH) .
3. The third file contains the distribution across the neighborhoods stratified by race/ethnicity. It states, given a race/ethnicity, the probability that an agent lives in any neighborhood in the city, and is derived from CDPH data.

Beyond these externally stored local data the model internalizes a set of parameters directly based on field data relating to the treatment effectiveness. The model holds the probability of being on PrEP at setup, based on observed rates in the RADAR data. It includes the adherence levels for PrEP based on a Atlanta based cohort [6] as local data for Chicago is not available. It contains rates of testing, and treatment, linkage to care, retention in care, and suppression, all based on CDPH HIV cascade data. And lastly, it has a parameter specifying for the population as a whole the likelihood of having a certain sex-role.

All this data is then used to set up a population of 6500 individuals, approximately representing the city of Chicago at a 1 : 10 scale. During this process we randomly sample 6500 individuals within the age range of 13 - 80 from the individuals file. This sampling process determines the agent population distribution stratified by age, race/ethnicity, HIV-status in during setup, and results in a population following distribution in Table 1.

	Blacks	Whites	Hispanic	Other	Population
Percentage of agents	29	37	28	6	100
HIV prevalence	44%	26%	22%	33%	30%

Table 1: Population distribution upon setup, in line with CDPH surveillance data

Additionally, during the set up, agents are assigned a more detailed set of attributes. They are assigned:

- An age in weeks based on their age by year, and randomly spread within that year.
- A sexual activity quintile, with a 20% chance to get assigned any of the quintiles.
- A tester? indicator, with 6.5% chance to not being a tester, a individual which will never go in for testing.
- Known HIV-positive individuals are assigned:
  - A date at which they contracted HIV, this is randomly chosen amount up to 333 weeks in the past. This guarantees that roughly 3% is in the acute HIV progression stage at setup.
  - A diagnosed?, indicator which is set to TRUE
  - A PLWH? indicator, which is set to TRUE
  - A suppressed? indicator, which is set to TRUE based on the rate per race/ethnicity type.

	Blacks	Whites	Hispanics	Others
Percentage PLWH suppressed	45.85%	51.97%	53.58%	60.92%

Table 2: The percentage of People living with HIV that are suppressed at setup, stratified by race/ethnicity type in line with CDPH surveillance data

  - A in-care? indicator that is set to TRUE, if indicated as suppressed, or FALSE otherwise.
- HIV-positive individuals that are unaware of their status are assigned:
  - A date at which they contracted HIV, this is randomly chose amount up to 333 weeks in the past. this guarantees that roughly 3% is in the acute HIV progression stage at setup in line with CDPH surveillance data.
  - Diagnosed?, PLWH?, in-care?, and suppressed? indicators that are all set to FALSE
- HIV-negative individuals:
  - Diagnosed?, PLWH?, in-care?, and suppressed? indicators that are all set to FALSE
  - A on-PrEP? indicator, which is set to TRUE based on the chance stratified by race/ethnicity type.
  - An in-care? indicator that is set to TRUE, if indicated as on-PrEP?, or as FALSE otherwise.
- A prep-adherence indicator and associated risk factor according to division in Table. 4

	Blacks	Whites	Hispanics	Others
PrEP at setup	7.7%	15.9%	10.1%	15.6%

Table 3: The percentage of HIV negative individuals on PrEP at setup, stratified by race/ethnicity type. Based on RADAR cohort data

	No adherence	Limited adherence	Moderate adherence	Full adherence
Meaning	No pills are taken	<2 pills per week	2-3 pills per week	4+ pills per week
Probability	22.1%	7.0%	10.0%	61.9%
Associated risk factor	1.00	0.69	0.19	0.95

Table 4: The PrEP adherence details at setup. Based on prior HIV modeling work [6]

- A sex-role indicator, with 18.3% being purely insertive, 22.1 % being purely receptive, and the remaining 59.6 % being versatile, based on RADAR cohort data
- A CCR5 mutation indicator, with Whites having a 17.6% chance of single mutation, and a 3.4% chance of double mutation. All other race/ethnicity types have a 2.1% chance of single mutation, and a 0.0% of double mutation, based on prior modeling work [6, 8].
- A circumcision? indicator, which is currently set to 76.8% for all ages and race/ethnicity types (based on [7]).
- A neighborhood of residence, based on their race/ethnicity in combination with the rates specified in the input file on neighborhood's racial compositions.

With all agents initialized LHM builts the network of sexual ties among them. To do so it runs the relationship formation process (which will be elaborated in detail in section 4.1.1) 104 times. This ensures mixing in the network and that any potential bias due to setup is sufficiently accounted for. More importantly,it ensures that the total number of ties in the network becomes stable, effectively indicating that the network is saturated with longer ties. Next, the model runs a single iteration of the one-night stand network formation process (as this completely resets every tick there is no path dependence we have to account for).

The burn-in period

A third step in setting up the model is to have the system self-correct for any potential biases that have been introduced during setup. e.g. in our setup of individuals, the HIV status is assigned independent of sexual behavior, while these attributes are clearly correlated. By letting the model run for a burn-in period, the dynamics of the model allow such misalignment to be slowly corrected, and consequently make those with high risk behaviors more likely to be HIV-positive. A check of model behavior (in terms of HIV incidence)

revealed that only the first year dynamics result in deviating outcomes, the following years the model produces consistent and stable results. To err on the safe side we opt for using a five-year of burn-in period, for which data will be discarded, that accounts any potentially remaining bias during set up.

With the model setup the details of the dynamics of the model and it processes are described in the next section that called "model behaviors and structure".

4 Model behaviors and structure

While the ODD standard for model reporting suggest reporting of all submodules as part of the details section of the document, earlier work by the authors [13] has highlighted that in high fidelity models the sheer amount of mechanisms and their interactions make it critical to provide additional overview of model behavior and dynamics. As such, this section will provide on overview of the structure of the behaviors in the model, prior to describing in detail how each of these functions and interact with other modules. The behaviors within the LHM, on the highest level, can be decomposed into two different components: A component that controls the partner network structure by network formation and dissolution, and a HIV spread component. Each of these operates relatively independently, where the network component provides the infrastructure on which the HIV spread component operates on. As such, the combination of the both components what drives system level behavior, and we elaborate each of these modules below.

4.1 Networks Of Sexual Partnerships

The agents, each representing an individual MSM, form sexual partnerships with one another based on their agent level preferences. Two types of such links are created; long-term relationships, which last anywhere beyond 1 week, or one-time ties which only last for a single week. This results in a long-term relationships network, and a one-time relationships network.

The rate at which links (of either type) are generated is based on preprocessed RADAR data and is directly fed into the model using two input files. In section 3.3.1 we elaborated how the field data is used to generate rates of link formation (per 6 months), and how these rates are stored respectively in the relation formation frequency file or the one-night-stand formation frequency file. While these rates vary by age, Race/ethnicity type, HIV-status, sex-role and activity quintile there are too many cross-sections to meaningfully provide descriptive statistics on these rates. We do however state that the average rate is 0.928 for long-term links to be formed in 6 months (with a minimum of 0 and a max of 7.6), and 0.867 for one-time

links (with a minimum of 0 and a max of 11.2). These rates per 6 months are consequently converted to an average weekly rates (stratified by Age, Race/ethnicity type, HIV-status, sex-role, and activity quintile). And each agent uses the appropriate rate as the mean of a Poisson distribution which is consequently sampled to determine the actual number of links formed in a given week by this agent.

4.1.1 Partnership formation

After having identified the number of new links that each agent wants to form in a given week, we describe how partnerships are formed. We illustrate this process for the long-term network only, as both types of networks are formed using the same process and logic, and only vary in the input data they use. We filter out all the agents that want at least one new link in a given time step (week) and combine them into a set of "tie-seekers". For long-term ties, in a given time step, we will have roughly 208 agents in the "tie-seekers" set (for one-time links this number is 189).

Next, we try to link the tie seekers to each other. To do so we have all tie seekers determine the characteristics of their desired next partner. Let the agent determining the partner characteristics be the ego. We determine the traits of the preferred partner for ego by filtering the relationship file for records that matches the ego's traits (Age, Race/ethnicity, HIV-status, and Sex-role). We then randomly sample a relationship record from among this filtered set, and set the desired traits (age, race/ethnicity, HIV-status and sex-role) of the next partner to be the traits of the alter described in the sampled record. We repeat this process for all tie seekers.

Next, we randomly select a single tie seeker and have it rank all other tie-seekers in terms of their match with the preferred partner traits by calculating a fitness score called the partner-score. This partner-score is calculated from the ego's perspective and is given by:

$$\text{partner-score} = 4/(\text{race-score}+\text{age-score}+\text{hiv-score}+\text{sex-score}),$$

where, age-score, race-score, hiv-score, and sex-score are computed as follows:

1. The **age-score** quantifies the compatibility between the ego's desired age for a partner and the potential partner's actual age. First determine if the partner age is within the margin of the ego's desired age: the default range is ± 2 years of the desired age. If the age is within this margin the age-related matching score is 1. If age is outside this range, the age-related matching score is equal to the squared age difference between the desired partner age and the actual partner age.

That is,

$$\text{age-score} = \begin{cases} 1, \text{ if } |\text{desired-age } - \text{ partner-age}| \leq 2 \\ (\text{desired-age } - \text{ partner-age})^2, \text{ otherwise} \end{cases}$$

where *desired-age* is the ego's desired age, and *partner-age* is the potential partner's age. Thus, the bigger the gap between the desired ages, the lower the partner-score will become.

2. The **race-score** quantifies the compatibility between the ego's desired race/ethnicity type for a partner, and the potential partner's actual race/ethnicity type. If the desired race is the same as the partner's race, the race-score is 1, otherwise the race-score is 50. That is,

$$\text{race-score} = \begin{cases} 1, & \text{if desired-race} = \text{partner-race} \\ 50, & \text{otherwise} \end{cases}$$

where *desired-race* is the ego's desired race, and *partner-race* is the potential partner's race. The weight of 50 was chosen to reflect the assumption we made that having a partner of an undesired race has a medium impact on the partner-score.

3. The **hiv-score** quantifies the compatibility between the ego's desired HIV status for a partner, and the potential partner's actual HIV status. If the desired HIV status is the same as the partner's HIV status, the hiv-score is 1, otherwise the hiv-score is 1000. That is,

$$\text{hiv-score} = \begin{cases} 1, & \text{if desired-hiv-status} = \text{partner-hiv-status} \\ 1000, & \text{otherwise} \end{cases}$$

where *desired-hiv-status* is the ego's desired HIV status, and *partner-hiv-status* is the potential partner's HIV status. This component of the partner score is used to capture the effect of sero-sorting on choosing potential partners. The weight of 1000 was chosen to reflect the assumption that HIV status plays a big role in choosing potential partners.

4. The **sex-score** quantifies the compatibility between the ego and the potential partner in terms of their sexual role preferences. As explained previously, sexual role of agents in the model fall under three categories: insertive, receptive, and versatile. For the matching to be compatible, one of the three following conditions must be satisfies. (i) The ego is insertive and the partner is receptive, (ii) the ego is receptive and the partner is insertive, (iii) either the ego or the partner is versatile. If any of these conditions are met, we will consider the ego and the partner to be compatible in term of sex role preferences, if not we will consider them to be incompatible. If the ego and the partner

are compatible, the sex-score is 1. Otherwise, the sex-score is 100000. That is,

$$\text{sex-score} = \begin{cases} 1, & \text{if compatible} \\ 100000, & \text{if incompatible.} \end{cases}$$

The weight of 10000 is chosen to indicate that sex role preferences plays a huge role in choosing potential partners, and that consequent matching should basically never occur.

Similarly, the ego asks the potential partners to calculate the partner-score from their perspective in the same way. That is, we calculate how well the ego matches the desired characteristics of the partner using the method outlined above. The eventual score for each dyad is then given by the multiplication of the partner-score from the ego's perspective and the partner-score from the partner's perspective. After computing the matching score for all potential links, the ego will use these scores as weights in a weighted random sample, and creates the link (and chooses a partner) that gets selected from a pull from this sample.

4.1.2 Partnership characteristics

Once an agent has chosen a partner, the characteristics of their relationship will be determined. Let whichever agent had the higher partner-score (the one for whom the relationship fitted best) be the ego. The characteristics of the relationship will be copied from on the characteristics of desired relationship that the ego wanted. These characteristics cover:

- The probability of condom usage per sex act in the relationship.
- The probability that ego will be the insertive party per sex act.
- The duration of the relationship in weeks.
- The mean number of sex acts per week.

After the characteristics of the relationship are determined, both the ego and the alter will have the number of ties they seek reduced by one. Either of these agents will remain in the tie-seekers pool as long as they still want more relationships that week, or will be taken out of the pool if they met their desired number of relationships for that week. After the first tie-seeker has created it's desired tie, a next tie-seeker is randomly selected from the pool, and goes through the same process. This process then continues until there are more than 2 tie seekers, and ties can be formed.
Apart from tie formation processes there are three additional processes that shape the network that is formed among the population of MSM in the model; partnership Dissolution, Becoming of age and Death.

4.1.3 Partnership dissolution

We have specified how ties are formed, yet, these ties do not remain permanently. As previously mentioned both one-time and long-term relationships have a prescribed duration. These durations are measured in weeks, and at each tick the remaining duration decreases by one, until it reaches zero. When the relationship's duration reaches zero, it will dissolve during the breaking relationship step in that tick. Each one-time tie will be dissolve the tick after it is created whereas the long-term ties persist from potentially much longer.

4.1.4 Becoming of age

After model initialization, there is ongoing process by which agents enter the model. Upon becoming of age of sexual onset, which in our model is assumed to be 13 years old, individuals have a chance to enter the model. We assume that there are no sexually active individuals younger than 13 years old and as such this population is excluded from the model. While the individuals under the age of 13 are excluded from the model, they are included in the "Individuals" input file, as this captures the whole population of Chicago; which includes individuals that are yet to become of age, and based on demographic trend, and even individuals that are yet to be born. As we have information regarding this population in our input file, we can keep track of all potential agents that become of age and hence should be considered to potentially be included in the model over time. To incorporate this becoming of age effect, at each time step (week), each individual in the population file that would turned 13 in the current year (e.g. in the first modeled year all 12 year old individuals are considered, in year second modeled year all those that are 11 year old are considered, and so on) has a probability of entering into the network. The weekly probability in our model is 0.1/52, which is based on the 1 : 10 scale of the population used in the model divided by 52 weeks per year.

4.1.5 Deaths

Similar to agents entering the model when they become of age, agent will leave the model when they are no longer at a sexual activate age, which in our model is assumed to be at age 80. As such, once agents reach an age of 80 years, they will exit the model at the beginning of the next tick. When leaving the model their ties will automatically dissolve.

On top of aging out of our target population agents can also be removed from the model by death. The exits due to mortality are modeled stochastically, and cover both mortality due to natural (non-HIV) causes and disease-induced mortality. Natural mortality rates (for the HIV negative individuals) are based on the Cook County 2014 all-cause death rates obtained from the CDC wondr webtool [14] and mortality rates for the HIV posi-

tive population are based on CDPH care continuum data over the period of 2014/2015. Both mortality rates are stratified by race and age and read into the model by means of pre-processed input files. Tables 5 and 6 show the probability of mortality per year by age and race for HIV positive and negative individuals respectively.AS these are annual probabilities of dying we divide these numbers by 52, to convert them to the probability of dying each week, which we then use as the mean in a poison process for each individual.

Age Range	Black	White	Hispanic	Other
13-19	0	0	0	0
20-29	0.018404908	0.003067485	0.003067485	0.003067485
30-39	0.036809816	0.012269939	0.003067485	0.003067485
40-49	0.058282209	0.039877301	0.027607362	0.003067485
50-59	0.049079755	0.04601227	0.021472393	0.003067485
60-80	0.042944785	0.030674847	0.018404908	0.003067485

Table 5: Probability of death for HIV-positive individuals CDPH care continuum data.

Age Range	Black	White	Hispanic	Other
< 15	0	0	0	0
15-19	0.002399	0.000353903	0.000698896	0
20-24	0.002709865	0.000990482	0.001294099	0
25-34	0.003179258	0.000918733	0.000996188	0.000293052
35-44	0.004159906	0.001498909	0.001465958	0.00066874
45-54	0.008689202	0.004523239	0.003404628	0.001848692
55-64	0.019064255	0.009606386	0.006724282	0.004765687
65-74	0.035637149	0.020151211	0.015084424	0.011250198
75-80	0.065105622	0.054741571	0.044151446	0.03271862

Table 6: Probability of death for HIV-negative individuals based on the Cook County 2014 all-cause death rates provided by the CDC via Wondr web portal [14].

4.2 HIV spread dynamics

With the network structure an its dynamics in place we have an infrastructure on which HIV can be transmitted. And while the network for-

mation component is hard to split up into smaller sub-modules, this HIV-transmission component easily be can further sub-divided. More specifically, we have to determine the number of sex acts, and the per act risk of transmission. The latter of which can be further sub-divided into several risk and protective factors that each have their own distinct impact on the per act risk of transmission. As such, we can consider each of these risk factors in relative isolation and include five categories of risk factors in our model; location based risk factors (a proxy for health and economic disparities), individuals attributes (such as CCR-mutations and circumcision), dyadic behavioral factors (such as condom use and sex-role), intra-host epidemiology factors (such a the viral-load), and care related factors (such as being suppressed and being on PrEP). Below we will discuss each factor affecting the risk:

4.2.1 Base risk

As with any disease we assume a base risks of transmission, in the case of HIV this risk is condition upon the sexual position of the HIV-positive partner. When the HIV-positive partner is receptive there is a 0.003379 probability of transmission per sex unprotected sex act, when the HIV-positive partner is receptive this probability is roughly 2.5 times as high, 0.008938 [15].

4.2.2 Circumcision of the HIV-negative partner

This base probability of transmission of HIV per sex act is further impacted by circumcision. If the HIV-negative partner is insertive and circumcised, the per act risk of transmission is reduced by a factor 2.5, (or the base risk is multiplied by 40%) [16]. Circumcision status is assigned to agents randomly when they are initialized into the model with a probability of 76.8% which is equal for all ages and race/ethnicity groups.

4.2.3 Intra-host epidemiology, viral-load of HIV-positive partner

The base per act probability of transmission is further condition upon the viral load, the number of copies of the HIV virus that is in 1 mL of blood, of the HIV-positive partner. More copies in one's blood make them more infectious, at a rate that is calculated as follows: $2.45^{(\text{viral-load}-4.5)}$, where the viral load is measured as the exponent of the number of copies in one's blood with a base of ten [17].

Viral-load does change over the course of an HIV infection, and to model this progression we (based on [18]) split the HIV progression process up in two stages: an acute stage, which describes the rapid rise and stabilisation of the viral load during initial infection, and lasts for 13 weeks after infection, and an stable stage which describes the remainder of the infection duration. For the acute stage we assume an average viral load of 6.0, whereas the viral

load for the stable stage is considered to be 4.5

Note that the above levels of viral load assumed no interventions or treatment, yet there is an effective Anti Retroviral Treatment (ART) available for HIV that suppressed the viral-load of individuals taking ART. As such, we include ART treatment as part of the viral-load calculations (more on how to get on ART is elaborated in section 4.3.7). More specifically, as it takes some time for the suppression the effects of ART to be completely achieved, we assume that being on ART reduces the viral load by 0.25 for each week it is maintained. As long as an individuals is on ART, their viral-load continues to decline, until it reaches a value of 1.5, which are effectively an undetectable level. If the individual discontinues ART treatment, viral load will within the next tick jump back to non-suppressed levels.

4.2.4 HIV-positive partner in the acute stage of HIV infection

The acute stage of the HIV infection (the first 13 weeks) presents an additional risk of transmission (beyond the increased viral load). As such, when in the HIV-positive partner has an HIV infection that is in the acute stage, the risk of transmission is increased by a roughly a factor of six [19]. We should be clear that this factor is based on a logistic model, and as such is both implemented and should be interpreted as a log-odd (with a value of ln(6)) rather than a factor.

4.2.5 CCR5 mutations of the HIV-negative partner

As mentioned in section 1.3 the mutation of the CCR5 allele makes individuals less susceptible for contracting HIV. Therefore when the HIV-negative person in a sero-discordant pair has a mutation of the CCR5 allele the risk of transmission for every act within that tie will be affected [8].

- When the HIV-negative person has a single mutation: the per act risk of transmission is reduced by roughly a factor 3.33, or more specifically by log-odds of ln(0.3) (as once more this value is derived from a logistic regression) for all acts within this tie.
- When the HIV-negative person has a double mutation: that person is effectively immune, which we operationalize by a log-odd of -100.

4.2.6 community level health disparities as a risk factor

In section 3.3.1 we discussed how various social factors and opportunities might lead to disparities that are geographically clustered in a city like Chicago. Racial segregation, poverty, access to healthcare and higher local prevalence of disease are but a few factors that might increase the per act risk of transmission locally. To capture such effects in our model we include a local risk inflation factor, as described in the "Generating a locally based

risk inflation factor" paragraph in section 3.3.1, resulting in local factors that range between 0.047520646 and 3.934317829. We assume these factors affect the risk of contracting HIV, and hence apply the risk inflation factor of the HIV-negative partner to the overall risk calculation within a given tie. We take the values provided in the risk inflation factor per neighborhood file and take the natural log of those and treat them as log-odds in our model.

4.2.7 Condom use

The per act risks of transmission presented above are based on unprotected sex acts, yet in many of the sex acts within the model a condom is used. For anal sex acts (which this model considers) the use of condoms is an significant protective factor, be it not nearly as effective as for vaginal sex. Using condoms in anal intercourse is associated with a risk reduction by roughly a factor 4 [20]. Here we again note that this factor is based on a logistic regression and as such is implemented as a log-odd (0.25) in our model.
The likelihood of condom use within a given tie is determined upon tie formation (see section 4.1.2), one of the parameters that is copied from the relationship data file upon creation is the likelihood of condom use. This likelihood is consequently used for every act within that tie to determine if a condom is or is not used.

4.2.8 PrEP use by the HIV-negative partner

Similar to condom use, the use of PrEP within a sero-discordant partnership can be a protective factor. PrEP can be prescribed to the HIV-negative partner, in section 4.3.2 we elaborate on this process. Yet, when the HIV-negative partner is on PrEP this will reduce the per act risk of transmission. The extend to which it does depends on the adherence levels of the HIV-negative person [21, 22].

- When the hiv-negative partner is non-adherent (22.1% of individuals): there is no risk reduction (log-odds of ln(1)).
- When the hiv-negative partner has limited adherence (7.0% of individuals): the per act risk is reduced by 31% (log-odds of ln(0.69)).
- When the hiv-negative partner has moderate adherence (10.0% of individuals): the per act risk is reduced by 81% (log-odds of ln(0.19)).
- When the hiv-negative partner has full adherence (61.9% of individuals): the per act risk is reduced by 95% (log-odds of ln(0.05)).

4.2.9 Calculating the per act risk of transmission

With all risk and protective factors identified we calculate the per act risk of transmission in the following manner:

1. We compute the "overall risk" given by

$$\text{absolute-risk} \;=\; \text{base-risk} \times \text{viral-risk} \times \text{circumcision-risk}.$$

2. We converting this overall risk to log-odds.

3. We add up the 5 the log-odds of all 6 factors (overall, acute, condom-use, CCR5, PrEP, and local) in the "combined" log-odds.

$$\text{log-total-risk} = \ln\left(\frac{\text{absolute-risk}}{1-\ \text{absolute-risk}}\right) + \ln(\text{acute-factor}) + \ln(\text{condom-factor})$$
$$+\ln(\text{prep-factor}) + \ln(\text{neighborhood-factor}) + \ln(\text{CCR5-factor}).$$

4. We convert the combined log-odds back to a probability:

$$\text{total-risk} \;= \frac{1}{1+\exp\left(-1\times\text{total-risk}\ \right)}.$$

We then do a single Bernoulli trial with this probability as the chance of successful transmission.

4.2.10 Rates of sexual activity

As each sex act is considered an independent Bernoulli trial, the observed HIV incidence each week is the product of both the per act risk of transmission and the number of acts that occur. As previously mentioned in section 4.1.2 the rates at which sex acts occur within a given tie is determined upon tie creation and based on field data from the relationship input file. This actual number of acts is then each week determined based on a Poisson distribution with that rate as a mean.

4.3 Treatment and Testing

One of the fundamental pieces of the LHM, directly linked to the aims of supporting decision making on interventions and policy development, are the treatment and testing dynamics. Which, both in the overall flow of the model as described by the pseudo code in section 1.5 and the per-act risks calculations in section 4.2 the treatment module (the presence of ART and PrEP), is presented as a prominent factor impacting model behavior. While it is evident that treatment, prevention, and testing dynamics can affect the spread of HIV, we have not yet elaborated how individuals within the model flow through the care cascade, therefore in this section we will highlight these dynamics.
Many paths by which individuals flow through the care system exist. Based on the cascade of HIV care we present the various pathways between the stages of the care that that are incorporated in our model. We identify

three main stages for both treatment (ART) and prevention (PrEP) care: being linked to care, being retained in care, being adherent to the care. An overview of the care process is presented in Figure 1, which highlights how individuals can transitions between these stages. Two things should be noted when considering this figure.
First; there is no explicit mention of adherence in the figure. For ART we simply do no have adherence data, however a proxy of adherence (for which we do have data) is captured by considering the suppressed state of individuals (suppression is considered the product of successful adherence to ART). For PrEP adherence we do have data, yet for the prevention arm of the care system (PrEP), adherence is considered an agent level behavior, independent of the care one receives. Clearly, one needs to be in care and on PrEP for adherence to have an effect, yet once this is the case we consider adherence of an individual to be determined by the the individual's attribute of adherence.
Second, each of the flows in the diagram is numbered, and represents a mechanism of transition among the states an individual can be in. Below we will use this numbering to describe the logic and parameterization within each transition in detail.

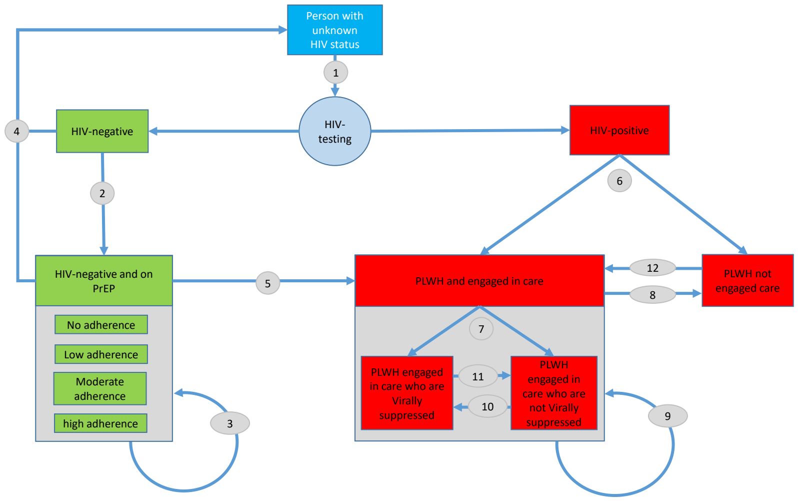

Figure 1: Levers of HIV model: Overview of treatment and prevention flows

4.3.1 Going in for HIV testing

From the individual's perspective interaction with the HIV care system starts by an individual getting a HIV test. The 2016 National HIV Behavioral Surveillance data (NHBS) [23] states that in Chicago 77.1% of the MSM indicated they were tested in the last 12 months. We convert this

annual testing percentage into an average daily rate of going in for testing among MSM. A 77.1% annual testing level corresponds to a 1/248 daily probability of going in for testing. Based on this rate of testing, we assume that every MSM will daily attempt to go in for testing, based on a Bernoulli process with a probability of success of 1/248. Each week seven of such trails are done (one for each day) and if any of these trails are successful the individual will go in for HIV testing that week. We assume that getting regular testing done is a part of the daily routine of most individuals that do not know their HIV status. A small group of individuals (6.5% of the population) will however never go in for testing.

4.3.2 PrEP assignment for those who test negatives for HIV

When tested negative for HIV, individuals can be linked to the care system and receive preventive care consisting of PrEP services. The goal of linkage to PrEP services is to have the individual start a PrEP regimen, which if adhered to significantly reduces the risk of infection. While the PrEP cascade identified various steps required to successfully initiate PrEP (such as informing, and prescribing), all such steps are subsumed in our model under the label linkage to PrEP.
Based on data gathered from the RADAR cohort study we know that this process in this Chicago based cohort resulted in approximately 11.7% of all HIV-negative individuals to be on PrEP. Combining this PrEP use with the rate of retention (see section below) we distill the rate of success for converting a negative HIV test into PrEP adoption to be 7%. As such, we assume that there is a 0.07 percent probability of being linked to PrEP care, and initiate a regimen, once tested negative for HIV.

4.3.3 Retention in PrEP care for those who are HIV-negative

Once individuals are on PrEP, there is a certain chance that individuals decide to terminate their use, and consequently drop out of care. From the RADAR cohort study we know that 53.5% of individuals on PrEP are still on prep a year later. We use this rate to model retention behavior to distill a weekly likelyhood of prescription renewal. PrEP is traditionally prescribed in regimens that have a limited duration of 13 weeks (3 months), which suggest that a care contact needs to occur every 3 months in order to retain an active prescription. Similar to the HIV testing rate we determine an average daily rate at which individual will need to have PrEP care contacts in order for 53.5% of individuals to remain on PrEP a given year. Figure 2 displays the probability of retention for each given return rate, and reveals that with an average rate 1 in 32 days we obtain a annual retention rates of 53.2%. This effectively means that every HIV-negative MSM on PrEP will do seven weekly Bernoulli trails with probability of success of 1/32, and if any of these trials is successful he will go in for care that week, get a new

HIV-test, be retained in care, and renews his PrEP prescription for another 13 weeks. Note that while this rate of going in for care is unrealistic for a given individual, it produces population dynamics that match field data.

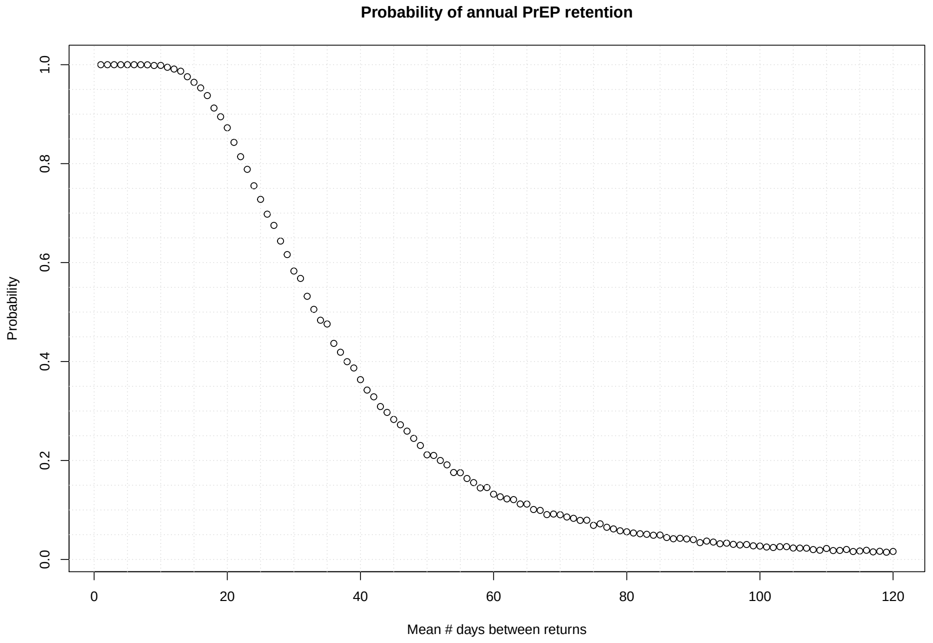

Figure 2: The chance of annual PrEP retention based on the rate of return visits (the mean number of days between returns)

4.3.4 Re-entering in care for those with unknown status

All individuals that are HIV-negative when dropping out of care are considered to return to the general MSM population. This includes those that are never linked to care, and those that have used PrEP prior and have fallen out of care. Their HIV-state can no longer be verified and as such they are considered a person with unknown HIV status and will behave as such, going (back) in for HIV-tests at the same rate as the overall population.

4.3.5 Testing positive for HIV while being on PrEP

A special case occurs when an individual on PrEP is tested positive during one of its care visits. Note that the effectiveness of PrEP as a protective factor is conditional upon an individual's adherence levels, and even under full adherence PrEP is not 100% effective. As such, be it in rare cases, one can be on PrEP and still get infected with HIV. In cases were such an infection is found, the newly infected individual will remain in care (he is still linked), but will be pushed to get on ART like any HIV-positive individual in care (with dynamics as will be described below).

4.3.6 Linkage to care for those who test HIV-positive

For the individuals that go in for their HIV-test and are found positive, we change their HIV-status to known HIV-positive, a state that individuals can never recover from. Next, we attempt to link these individuals to care. We follow a similar logic as for those testing negative, in that we assume that doing the initial HIV-test is no indicator of being linked to care. Instead we only assume individuals to be in care when they get a CD4 viral load after initially being tested positive. Based on HIV-care cascade data obtained from CDPH we know that for MSM in Chicago in the large majority of the cases linkage indeed occurs (see Table 7).

	Blacks	Whites	Hispanic	Other
Ages 13 to 33	87.70%	92.05%	91.68%	86.09%
34 y/o and over	87.69%	93.87%	94.20%	93.85%

Table 7: Probabilities of linkage to care after a positive HIV test, based on CDPH HIV care cascade data.

4.3.7 Getting suppressed for HIV-positives whom are linked to care

Once HIV-positive individuals are linked to care we assume that all of them are put on ART and have their viral-load suppressed. For various reasons such suppression efforts can be ineffective, among which: failure to actually prescribe ART,and various biomedical factors. However, the predominant reason for failure to become suppressed will be a failure to adhere to medication by the individual. We do not distinguish between these causes but consider adherence to be the driver of viral suppression. We capture the rate at which HIV-positive individuals in care will become suppressed based on the data obtained from the CDPH, and find that the likelihood that individuals do become suppressed after their initial linkage varies across race/ethnicity and age (see Table 8).

	Blacks	Whites	Hispanic	Other
Ages 13 to 33	42.63%	57.54%	50.79%	45.98%
34 y/o and over	44.54%	60.68%	58.30%	42.11%

Table 8: Probabilities of becoming suppressed after the initial care visit, based on CDPH HIV care cascade data.

4.3.8 Rates of going in for treatment for in care HIV-positives

Similar to the HIV-negative in-care population, being in care and being HIV-positive changes the daily care routines of the individual. Based on the CDPH care cascade data we determine exactly how these routines change

once in care. Based on the average days between testing in our field data, we determine the rate at which individuals go in for treatment once diagnosed. These rates are presented in Table 9 below.

	Blacks	Whites	Hispanic	Other
Ages 13 to 33	1/94	1/91	1/90	1/81
34 y/o and over	1/77	1/98	1/83	1/74

Table 9: Daily probabilities of going in for a care visit for HIV-positive individuals, based on CDPH HIV care cascade data.

Individuals will intend to go in for treatment based on a daily Bernoulli trial with the associated probability as the chance of success. Similar to regular HIV-testing, 7 of these trial will be done each week, and if any of them is successful the individual will intent to go in for treatment that week.

4.3.9 Dropping out of care for HIV-positives

Once the intend to go in for testing is determined in a given week, we determine the extend to which the visit in fact takes place. In contrast to the PrEP retention mechanism, for ART there is no limited duration of medication. As such there is no temporal threshold for being retained in care. Instead we account for the mechanism of dropping out of care by means of a probabilistic process that occurs every time there is an intend to go in for testing/treatment.

The field data from CDPH covers information for up to 4 care contacts over the first two years after diagnosis (the first of which refers to linkage to care). Based on this data we determine the rate of dropping out in each of the consequent care visits after linkage (table 10). We assume that after three care visits, individual has become used to their new reality of being HIV-positive, and as such assume the drop-out behavior to remain stable after these initial visits.

	1st care visit	2nd care visit	3rd care visit (and beyond)
Blacks, ages 13 to 33	13.69%	12.80%	11.61%
Whites, ages 13 to 33	11.08%	11.65%	12.50%
Hispanics, ages 13 to 33	10.23%	7.04%	10.02%
Other, ages 13 to 33	10.43%	13.04%	9.57%
Blacks, 34 y/o and over	16.22%	8.71%	7.21%
Whites, 34 y/o and over	11.70%	10.58%	10.31%
Hispanics, 34 y/o and over	9.06%	6.16%	7.61 %
Other, 34 y/o and over	6.15%	6.15%	10.77%

Table 10: Probabilities of dropping out of in care (1 - rate of retention), based on CDPH HIV care cascade data.

Note that persistent dropout rate of roughly 10% for all care visits beyond the 2nd dictates that all individuals at some point will be dropping out of care. These individuals can re-enter care, a process that is described in section 4.3.12.

4.3.10 Suppression during retention in care

Similar to the initial care visit, the rates of suppression while being retained in care can be determined. Our field data allows us to determine the probability that individuals that did not initially become suppressed in previous visits will do so after the 2nd, 3rd, and later care visits. We follow a similar logic to the retention/drop out behavior, in that we assume the that patterns after the latest care visit will continue indefinitely. The rates of suppression (including those after the first visit) are captured in Table 11. Note that these rates are cumulative and describe the chance of suppression of an individual while being retained over multiple visits.

	1st care visit	2nd care visit	3rd care visit (and beyond)
Blacks, ages 13 to 33	42.63%	23.34%	9.84%
Whites, ages 13 to 33	57.54%	21.14%	7.31%
Hispanics, ages 13 to 33	50.79%	22.85%	11.46%
Other, ages 13 to 33	45.98%	17.91%	19.72%
Blacks, 34 y/o and over	44.54%	26.75%	10.87%
Whites, 34 y/o and over	60.68%	21.42%	7.44%
Hispanics, 34 y/o and over	58.30%	17.85%	12.18%
Other, 34 y/o and over	42.11%	25.82%	5.99%

Table 11: Cumulative probabilities of becoming suppressed a care visit, out of in care (1 - rate of retention), based on CDPH HIV care cascade data.

4.3.11 Becoming unsuppressed while retained in care

Here we introduce a mechanism for virally suppressed individuals to no longer be suppressed while they are still being retained in care. This is a plausible scenario for individuals who do not strictly adhere to their ART medications and care visits even though they technically remain in care. The probability of becoming unsuppressed is the same for every suppressed individual in care. There was no field data available to us regarding this parameter of being unsuppressed, so we calibrated our model using two observables to match the field data. This is the only parameter in our model for which we used calibration instead of using field data directly. To be more precise, we found the probability of being unsuppressed that minimizes the

following weighted mean squared error,

$$\text{wmse} = \left(\frac{m_d - a_d}{a_d}\right)^2 + \left(\frac{m_c - a_c}{a_c}\right)^2,$$

where m_d is the modeled ratio of suppressed individuals among those diagnosed HIV+. We know from CDPH surveillance data that in 2015 $a_d = 0.515$, the actual ratio of suppressed individuals among those diagnosed HIV+. Similarly, m_c is the modeled ratio of suppressed individuals among those in care, and from field data we know $a_c = 0.7961$, the actual ratio of suppressed individuals among those in care. To find this parameter that minimizes the above error, we used a tool in NetLogo called BehaviorSearch that uses genetic algorithms to search thorough a parameter space in order to optimize model outcomes as desired [24]. After getting an approximate value for this parameter using BehaviorSearch, we performed an exhaustive search to find the exact value up to four decimal points that minimizes the error above. As a result, we found the probability of becoming unsuppressed to be prob-unsuppressed = 0.0044.

4.3.12 Re-entering into care for known-HIV-positives

Those HIV-positive individuals that fail to be retained or linked to care, will not return to the regular population. Instead they will be carry a "known-HIV-positive" indicator, as they have been previously diagnosed, which is combined with an indicator of the stage at which they have fallen out of care; either "failed retention" or "failed to link". We assume that these individuals will attempt to re-enter treatment at a same rate that is different from the general population, which goes in for HIV testing with an average rate of once every 248 days. We assume that for those with a known HIV-positive status the inclination of reengaging into care is half of that of the general population, so these individuals will reattempt linkinge at a rate of 1/496 days.

5 Observations and model alignment

The previous sections have described the Levers of HIV model, the individual level behaviors in it, and its parametrization. We have highlighted how behaviors have been anchored using field data, and as such should enable the model to be an accurate representation of the HIV spread dynamics in the real world. To test this assumption in this section we take a closer look at the system level behaviors of the model. As the aim of the model is support decision making towards attaining the getting to zero goals of 90% reduced incidence by the year 2030, we focus our efforts in validation on the incidence of new HIV cases.

We do not simply want to track the number of incidences in the model, we also want to know how they are distribution, as these are know to vary

both by race and age and their cross-sections. Such distributions are critical in understanding how disparities emerge and are potentially impacted by various policy interventions. Consequently we track the distribution of the incidence cases along these dimensions throughout our simulations. We track incidence in the LHM by recording for each year;

1. The cumulative number of new incidence cases
2. The cumulative number of new incidence cases stratified by race/ethnicity
3. The cumulative number of new incidence cases stratified age (in years)

We measure these observations over a period of 15 years (after the burn-in), representing the period of 2016 to 2030.

While the incidence emerges from a stochastic process with relatively low frequency, relatively strong variance in the incidence at any given time-step are to be expected. Aggregating incidence over the course of a year negates some of this variance, however both path dependence within a given simulation run as well as remaining variance requires each scenario to be repeated multiple times to get draw reliable conclusions. To determine the number of repetitions required to obtain reliable results, we ran a set of 250 initial repetitions of the baseline model. Based on these initial results, or more specifically the variance of the cumulative incidence numbers in them, we determined that a total of at least 44 repetitions would be needed to enable us to provide the mean behavior with an accuracy of +/- 50 cases, at a confidence interval of 95%.
While mean values are informative we additionally check the distribution of modeled outcomes by introducing a 80% simulation prediction interval. This interval corresponds to a central range of one-year age-specific incidences based on the 44 simulations (i.e., the 10th and 90th prediction percentiles over the 44 replicates of a given scenario).

5.1 Validation based on incidence numbers

Note that our model as been parameterized based on 2015 data from various sources. As such to validate our model predictions we can compare the modeled data to field data currently available for 2016. Based on HIV cascade data from CDPH (Table 12) we know that 665 new HIV incidence cases have been identified among MSM in Chicago in 2016, a number we can compare to the prediction of the LHM outcomes.

Note that CDPH data captures, and thus reports, the ***diagnosed*** new incidence cases, as new cases not diagnosed are unknown to them and hence cannot counted. Our model however counts ***all*** new incidences cases. As such, to align the reported outcomes, we corrected the modeled outputs

Age Group	2016 Field bfdata (CDPH)	Modeled prediction: Mean	modeled Prediction: 80% simulation prediction interval	modeled Prediction: 95% confidence interval of the mean
13-19	46	93.1	44.0 - 149.0	80.6 - 105.5
20-29	313	273.2	176.4 - 370.8	249.2 - 297.3
30-39	159	172.2	111.7 - 234.0	157.6 - 186.7
40-49	79	83.2	53.1 - 112.8	75.5 - 90.9
50-59	55	39.9	20.0 - 59.4	35.1 - 44.6
60 +	13	23.1	9.8 - 36.1	18.5 - 25.8
Total	665	690.8	519.7 - 827.7	647.8 - 733.7

Table 12: This table shows the actual newly identified incidence cases stratified by race/ethnicity type and age group

to match the meaning to the CDPH data required. Based on data from CDC [25] we add a probability of being diagnosed for each modeled incidence case (see Table 13). Furthermore we need to adjust for the scale of the model, we only model 10% of the population, and hence need to increase our results by a factor of 10 to scale them up to the full city. Note that the population size stays roughly the same but does in fact vary in our model, consequently the actual scaling is determined based on the actual population size in the model at time of the observation. After these two adjustments the numbers presented in the model are conceptually the same as those reported by CDPH.

Age group	Probability of diagnosis (%)
13 - 19 years old	55.6
20 - 29 years old	70.9
30 - 39 years old	84.5
40 - 49 years old	91.0
50 - 59 years old	92.9
$>$ 60 yeas old	93.7

Table 13: This table shows the probability that an HIV infection is diagnosed, stratified by age group. These values are taken from HIV CDC surveillance supplemental report in 2017 (page 55) [25].

After implementing the alignments above, the mean number of incidence cases for 2016 predicted by the model is 690.8 (with a 95% confidence interval of 647.8 - 733.7, and 80% simulation prediction interval of 519.7 - 827.7). While the mean slightly over estimates of the actual reported incidences we are very close to reproducing the originally observed dynamics and the incidence number in the real world. This is an indication of our model living up to its aim of being a high fidelity model, capturing nuances of real world behaviors accurately.

5.2 Validation based incidence distributed over age

When considering the distribution of the HIV incidence cases over age in Table 12 one can see that our model is relatively close to the originally observed incidence distributions by age, even without any prior assumptions on how incidence should be distributed over age. We do find that the LHM on average is slightly underestimating incidences in the 20-29 age bracket and overestimating the tails of the distribution, the latter could well be the result of our somewhat crude way of extrapolation for older ages, as described in Section 3.3.1.

5.3 Validation based on incidence distributed over race/ethnicity type

Lastly, we consider the distribution of incidence by race/ethnicity type. Based on a combination of demographic information and incidences numbers we can determine the per person incidence rates in both the real world and the LHM. These rates are presented in Table 14 below. These results clearly highlight the misalignment that would persist in a model without the implementation of community based health disparity module impacting transmission risks. What it more is shows that even with such dynamics present some discrepancies between modeled and observed rates remain.

	**NH Black**	**NH White**	**Hispanic**	**Other**
**Observed values (based on CDPH data)**				
Incidence rate per 10k	183.26	51.76	91.78	47.75
**LHM <u>without</u> the Health disparity Module**				
80% Prediction Interval	57.14 - 122.06	65.99 - 117.13	70.09 - 142.82	21.24 - 122.33
Mean incidence rate	88.47	89.72	109.08	68.22
Relative error	51.72%	73.35%	18.85%	42.89%
**LHM <u>with</u> the Health disparity Module**				
80% Prediction Interval	110.47 - 202.5	42.85 - 74.01	75.97 - 147.95	20.70 - 103.48
Mean incidence rate	153.36	56.04	114.05	62.56
Relative error	-16.31%	8.27%	24.27%	31.02%

Table 14: The rates of HIV incidence by race/ethnicity group

6 Interventions

We described the aims of the Levers of HIV Model as supporting the decision making by local health departments in determining how to allocate their services. To achieve this goal we can model the effects of various interventions the health department could implement and compare their impact to get a relative sense of the sensitivity of the HIV system to such perturbation. Based on the HIV care system we identify six targets for intervention, three related to PrEP and three related to ART treatment module: PrEP

linkage, PrEP retention, PrEP adherence, ART linkage, ART retention and ART adherence. Without going into detail about *how* to implement such intervention we explore the impact of potential perturbations in each of these levers.

6.1 PrEP linkage

PrEP linkage refers to the likelihood that a person with a negative HIV test will be put on PrEP. As such, it compounds the actual linkage to care and the start of PrEP medication into a single rate. The default rate at which this occurs is estimated based on the rate of retention on PrEP and the persistence of PrEP usage within the RADAR cohort study. Under the observed rate of retention we found that a PrEP linkage rate of 7% yields an overall PrEP usage rate that is equal to what we observe in our data (12% of all eligible individuals being on PrEP).
We assume that any attempt to improve the linkage to PrEP will take some time to take effect. As such, it seems more realistic to think of such interventions as an annual increase by a certain percentage, rather than a instant one-of change in rate. For this reason we implement interventions in PrEP linkage as an annual increase of the rate of PrEP linkage increases. Instead of the baseline scenario with a flat 7% linkage rate, the intervention scenarios with have a rate that increases over time. To cover the various strengths of interventions in the linkage to PrEP we adopt a total of six scenarios for PrEP linkage:

1. Scenario 0 (baseline): which has a base PrEP linkage of 7% that remains stable over time
2. Scenario 1 (minimal): Which has a base linkage of 7% that increase every year by 2%, resulting in 37% linkage to PrEP by 2030.
3. Scenario 2 (minor): Which has a base linkage of 7% that increase every year by 4%, resulting in 67% linkage to PrEP by 2030.
4. Scenario 3 (moderate): Which has a base linkage of 7% that increase every year by 6%, resulting in 97% linkage to PrEP by 2030.
5. Scenario 4 (extreme): Which has a base linkage of 7% that increase every year by 8% resulting in 100% linkage to PrEP from 2029 onwards.
6. Scenario 5 (max): Which has a base linkage of 7% that increase every year by 10%, resulting in 100% linkage to PrEP from 2026 onwards.

6.2 PrEP retention

PrEP retention is measured in the percentage of PrEP users that one year after initiation are still on PrEP. From the RADAR data we distilled that 53.5% of PrEP users are still on PrEP a year later. Based on the retention

we assume a uniform rate of discontinuing PrEP (and falling out of care), a rate which we modify to capture the efforts made to increase the retention of individuals on PrEP.
To cover the various strengths of interventions in the retention of PrEP we adopt a total of four scenarios for PrEP retention:

1. Scenario 0 (baseline): In which 53.5% of individuals are retained each year(rate of going in for care on average every 32 days).
2. Scenario 1 (minor): In which 65.0% of individuals are retained each year (rate of going in for care on average every 28 days).
3. Scenario 2 (moderate): In which 75.0% of individuals are retained each year (rate of going in for care on average every 24 days).
4. Scenario 3 (extreme): In which 85.0% of individuals are retained each year (rate of going in for care on average every 21 days).

6.3 PrEP adherence

PrEP adherence refers to the level of adherence to the medication, the amount of pills an individual takes while being prescribed PrEP, and measures the extent to which PrEP is effective. Four levels of adherence have been identified:

- No-adherence: No pills are taken, 22.1% of individuals are non-adherent, with 0% risk reduction
- Low-adherence: < 2 pills per week, 7.0% of individuals take < 2 pills per week, with 31% risk reduction
- Moderate-adherence: 2-3 pills per week, 10.0% of individuals take 2-3 pills per week, with 81% risk reduction
- Full-adherence: 4+ pills per week, 61.9% of individuals take 4+ pills per week, with 95% risk reduction

We refer to this baseline scenario as 22.1/7.0/10.0/61.9 referring to the likelihood of individuals having either of the four adherence level.
When considering interventions in the PrEP adherence, we refer to any activity that might cause a individuals to move from one adherence level towards a higher level of adherence. To cover the various strengths of interventions in the adherence of PrEP we adopt a total of three scenarios for PrEP adherence:

1. Scenario 0 (baseline): In which the distribution among adherence levels is 22.1 / 7.0 / 10.0 / 61.9 respectively, resulting in an average risks reduction of 69.1%.

2. Scenario 1 (moderate): Ensuring no more no-adherence. The percentage of non-adherents is set to 0 and these individuals are distributed evenly across the other levels of adherence. So the distribution among adherence levels is 0 / 14.4 / 17.4 / 69.2 respectively, resulting in an average risks reduction of 84.3%.

3. Scenario 2 (max): Ensuring full adherence. All individuals are assumed to be fully adherent, this is the maximum efficiency PrEP medication can achieve (effectiveness remains conditional on effective linkage and retention), so the distribution among adherence levels is 0 / 0 / 0 / 100 respectively, resulting in an average risks reduction of 95.0%.

6.4 ART linkage

Linkage to ART care refers to the rate by which individuals that get a positive HIV test obtain a CD4 viral load test. Once they have had their CD4 viral load tested we consider them linked to care and assume they will be put on ART from that point onward. We note that the effectiveness of this ART regiment can vary (see ART adherence). Based on CDPH data we distilled that ART linkage success rates are relatively high and vary between 86%-95% (see table 7) conditional on age-group and race/ethnicity. When considering interventions in ART linkage we consider increasing the rate of successful linkage across all these age and race/ethnicity groups equally (although specific effort could be implemented). While the linkage percentages are already extremely high, we expect limited impact of the level on systemic behaviors, as such we see little value in subdividing the spectrum of potential interventions in too many levels. While we do want to provide a complete overview we cover the full the spectrum of interventions by introducing two scenarios for ART adherence:

1. Scenario 0 (baseline): Linkage percentage per sub-group are specified in 7.

2. Scenario 1 (max): The baseline linkage percentages are increased by 14% so that all sub-groups are always linked.

6.5 ART retention

ART retention refers to the extent to which individuals that are in ART care, stay in care. It is measured based on the rates and success by which individuals are returning for their care visits (based on CDPH data). Based on these rates each individual in ART care has a probability of dropping out of care at each attempt to go in for a ART care visit. We determined that the drop-out rates are roughly 12% (between 6 and 15% depending on the retention in care, age group and race/ethnicity (see Table 10)).
When we consider interventions in ART retention we consider interventions

that effectively reduce the rate at which individuals drop out of care, and implement such effects by reducing the drop-out rates in Table 10 by $x\%$ across the board (so in all consecutive visits for each age race/ethnicity group). To cover the various strengths of interventions in the retention of ART care we adopt a total of four scenarios for ART retention:

1. Scenario 0 (baseline): In which drop-out rates per visit are according to those presented by CDPH data (Table 10), resulting in 72.4% being retained for 3 care visits

2. Scenario 1 (minor): In which the baseline drop-out rates are reduced by 2% across the board, resulting in 77.4% being retained for 3 care visits

3. Scenario 2 (moderate): In which the baseline drop-out rates are reduced by 5% across the board, resulting in 85.2% being retained for 3 care visits

4. Scenario 3 (extreme): In which the baseline drop-out rates are reduced by 10% across the board, resulting in 96.7% being retained for 3 care visits.

6.6 ART adherence

ART adherence refers to the success of the ART regiment. More specifically, it refers to the rate at which individuals become suppressed (by means of ART) when they are in care. Based on field data we have observed that the longer one remains in care, the more likely one is to become suppressed. As such suppression slowly increases over care visits, and can be considered as cumulative chance over care visits. Where the suppression is roughly 50% after the 1st visit, another 25% (or 50% of those still unsuppressed) get suppressed after the 2nd, and another 10% (or 40% of the remaining unsuppressed) thereafter ((conditional on the age and race/ethnicity, see Table 11 for details). When we consider interventions in the ART adherence we consider probability of becoming suppressed at each consecutive care visit e.g. the 50%, 50% and 40% probabilities mentioned above. We implement interventions as perturbations to these probabilities, and similar to the PrEP linkage assume that such changes will take place by a gradual process. As such, during intervention we increase the probabilities annually by a given amount. To cover various strengths of interventions in the adherence to ART and the consequent viral suppression we adopt a total of four scenarios for ART adherence:

1. Scenario 0 (baseline): The suppression is in line with the rates presented in Table 11.

2. Scenario 1 (minor): The suppression probabilities for each visit are increased by 2% annually. This means that after nine years roughly 68%

gets suppressed after their first visit and roughly 95% is suppressed after their third visit.

3. Scenario 2 (moderate): The suppression probabilities for each visit are increased by 3% annually. This means that after six years roughly 63% gets suppressed after their first visit and roughly 95% is suppressed after their third visit.

4. Scenario 3 (extreme): The suppression probabilities for each visit are increased by 5% annually. This means that after four years roughly 70% gets suppressed after their first visit and roughly 95% is suppressed after their third visit.

Based on the number of levels in each of the intervention levers our experiment results in 2304 ($6*4*3*2*4*4 = 2304$) different intervention scenarios.

References

[1] Grimm V, Berger U, Bastiansen F, Eliassen S, Ginot V, Giske J, et al. A standard protocol for describing individual-based and agent-based models. Ecological Modelling. 2006 Sep;198(1-2):115–126. Available from: https://linkinghub.elsevier.com/retrieve/pii/S0304380006002043.

[2] Grimm V, Berger U, DeAngelis DL, Polhill JG, Giske J, Railsback SF. The ODD protocol: A review and first update. Ecological Modelling. 2010 Nov;221(23):2760–2768. Available from: https://linkinghub.elsevier.com/retrieve/pii/S030438001000414X.

[3] Grimm V, Railsback SF, Vincenot CE, Berger U, Gallagher C, DeAngelis DL, et al. The ODD Protocol for Describing Agent-Based and Other Simulation Models: A Second Update to Improve Clarity, Replication, and Structural Realism. Journal of Artificial Societies and Social Simulation. 2020;23(2):7. Available from: http://jasss.soc.surrey.ac.uk/23/2/7.html.

[4] NASTAD. Ending the HIV Epidemic: Jurisdictional Plans;. Available from: https://nastad.org/resources/ehe-plans-and-websites.

[5] Grey JA, Bernstein KT, Sullivan PS, Purcell DW, Chesson HW, Gift TL, et al. Estimating the Population Sizes of Men Who Have Sex With Men in US States and Counties Using Data From the American Community Survey. JMIR Public Health and Surveillance. 2016 Apr;2(1):e14. Available from: http://publichealth.jmir.org/2016/1/e14/.

[6] Jenness SM, Goodreau SM, Rosenberg E, Beylerian EN, Hoover KW, Smith DK, et al. Impact of the Centers for Disease Control's HIV Preexposure Prophylaxis Guidelines for Men Who Have Sex With Men in the United States. Journal of Infectious Diseases. 2016 Dec;214(12):1800–1807. Available from: https://academic.oup.com/jid/article-lookup/doi/10.1093/infdis/jiw223.

[7] Owings M, Uddin S, Williams S. Trends in Circumcision Among Male Newborns Born in U.S. Hospitals: 1979–2010. 2019 Jun;Available from: https://www.cdc.gov/nchs/data/hestat/circumcision_2013/circumcision_2013.htm.

[8] Marmor M, Sheppard HW, Donnell D, Bozeman S, Celum C. Homozygous and HeterozygousCCR5-32 Genotypes Are Associated With Resistance to HIV Infection. JAIDS Journal of Acquired Immune Deficiency Syndromes. 2001 Aug;27(5):472–481. Available from: https://journals.lww.com/jaids/Abstract/2001/08150/Homozygous_and_Heterozygous_CCR5__DELTA_32.9.aspx.

[9] Mustanski B, Birkett M, Kuhns LM, Latkin CA, Muth SQ. The Role of Geographic and Network Factors in Racial Disparities in HIV Among Young Men Who have Sex with Men: An Egocentric Network Study. AIDS and Behavior. 2015 Jun;19(6):1037–1047. Available from: http://link.springer.com/10.1007/s10461-014-0955-0.

[10] Mustanski B, Morgan E, D'Aquila R, Birkett M, Janulis P, Newcomb ME. Individual and Network Factors Associated With Racial Disparities in HIV Among Young Men Who Have Sex With Men: Results From the RADAR Cohort Study. JAIDS Journal of Acquired Immune Deficiency Syndromes. 2019 Jan;80(1):24–30. Available from: https://journals.lww.com/00126334-201901010-00004.

[11] Wilensky U. NetLogo. Center for Connected Learning and Computer-Based Modeling, Northwestern University, Evanston, IL. 1999;Available from: http://ccl.northwestern.edu/netlogo/.

[12] Chicago Department of Public Health. HIV STI Surveillance Report 2020. 2020;p. 60. Available from: https://www.chicago.gov/content/dam/city/depts/cdph/statistics_and_reports/CDPH-HIV-STI-REPORT-2020.pdf.

[13] Vermeer W, Hjorth A, Jenness SM, Brown CH, Wilensky U. Leveraging Modularity During Replication of High-Fidelity Models: Lessons from Replicating an Agent-Based Model for HIV Prevention. Journal of Artificial Societies and Social Simulation. 2020;23(4):7. Available from: http://jasss.soc.surrey.ac.uk/23/4/7.html.

[14] Centers for Disease Control and Prevention. CDC WONDER;. Available from: https://wonder.cdc.gov/.

[15] Vitinghoff E, Douglas J, Judon F, McKiman D, MacQueen K, Buchinder SP. Per-Contact Risk of Human Immunodificiency Virus Tramnsmision between Male Sexual Partners. American Journal of Epidemiology. 1999 Aug;150(3):306–311. Available from: https://academic.oup.com/aje/article-lookup/doi/10.1093/oxfordjournals.aje.a010003.

[16] Gray RH, Kigozi G, Serwadda D, Makumbi F, Watya S, Nalugoda F, et al. Male circumcision for HIV prevention in men in Rakai, Uganda: a randomised trial. The Lancet. 2007 Feb;369(9562):657–666. Available from: https://linkinghub.elsevier.com/retrieve/pii/S0140673607603134.

[17] Wilson DP, Law MG, Grulich AE, Cooper DA, Kaldor JM. Relation between HIV viral load and infectiousness: a model-based analysis. The Lancet. 2008 Jul;372(9635):314–320. Available from: https://linkinghub.elsevier.com/retrieve/pii/S0140673608611150.

[18] Little SJ, McLean AR, Spina CA, Richman DD, Havlir DV. Viral Dynamics of Acute HIV-1 Infection. Journal of Experimental Medicine. 1999 Sep;190(6):841–850. Available from: https://rupress.org/jem/article/190/6/841/29908/Viral-Dynamics-of-Acute-HIV1-Infection.

[19] Leynaert B, Downs AM, de Vincenzi I, for the European Study Group on Heterosexual Transmission of HIV. Heterosexual Transmission of Human Immunodeficiency Virus: Variability of Infectivity throughout the Course of Infection. American Journal of Epidemiology. 1998 Jul;148(1):88–96. Available from: https://academic.oup.com/aje/article-lookup/doi/10.1093/oxfordjournals.aje.a009564.

[20] Varghese B, Maher JE, Peterman TA, Branson BM, Steketee RW. Reducing the Risk of Sexual HIV Transmission: Quantifying the Per-Act Risk for HIV on the Basis of Choice of Partner, Sex Act, and Condom Use. Sexually Transmitted Diseases. 2002 Jan;29(1):38–43. Available from: http://journals.lww.com/00007435-200201000-00007.

[21] Grant RM, Lama JR, Anderson PL, McMahan V, Liu AY, Vargas L, et al. Preexposure Chemoprophylaxis for HIV Prevention in Men Who Have Sex with Men. New England Journal of Medicine. 2010 Dec;363(27):2587–2599. Available from: http://www.nejm.org/doi/abs/10.1056/NEJMoa1011205.

[22] Grant RM, Anderson PL, McMahan V, Liu A, Amico KR, Mehrotra M, et al. Uptake of pre-exposure prophylaxis, sexual practices, and HIV incidence in men and transgender women who have sex with men: a cohort study. The Lancet Infectious Diseases. 2014 Sep;14(9):820–829. Available from: https://linkinghub.elsevier.com/retrieve/pii/S1473309914708473.

[23] Centers for Disease Control and Prevention. HIV Infection Risk, Prevention, and Testing Behaviors among Men Who Have Sex With Men—National HIV Behavioral Surveillance, 20 U.S. Cities, 2014. 2016;(15):32. Available from: https://www.cdc.gov/hiv/pdf/library/reports/surveillance/cdc-hiv-surveillance-special-report-number-15.pdf.

[24] Stonedahl F, Wilensky U. Finding Forms of Flocking: Evolutionary Search in ABM Parameter-Spaces. In: Bosse T, Geller A, Jonker CM, editors. Multi-Agent-Based Simulation XI. vol. 6532. Berlin, Heidelberg: Springer Berlin Heidelberg; 2011. p. 61–75. Series Title: Lecture Notes in Computer Science. Available from: http://link.springer.com/10.1007/978-3-642-18345-4_5.

[25] Centers for Disease Control and Prevention. Monitoring Selected National HIV Prevention and Care Objectives by Using HIV

Surveillance Data—United States and 6 Dependent Areas, 2017. 2019;24(3):74. Available from: http://www.cdc.gov/hiv/library/reports/hiv-surveillance.html.
