# Supplementary material for: Agent-based model projections for reducing HIV infection among MSM: Prevention and care pathways to end the HIV epidemic in Chicago, Illinois
Source: PLoS One. 2022 Oct 17;17(10):e0274288. doi: 10.1371/journal.pone.0274288 (PMC9576079; doi:10.1371/journal.pone.0274288)
Supplement: S2 Appendix — This appendix describes in detail the Bayesian methods used for prediction in this paper. (PDF) [file pone.0274288.s002.pdf]

Explanation of a Simulation Method to Obtain a Posterior Predictive Distribution of the 10-Year HIV Incidence for MSM in Chicago Based on Current Progress

immediate

March 11, 2022

The supplementary information section provides a definition, notation and references for calculating the posterior predictive distribution using simulation modeling. We define the random variable R_{2030} as the number of incidence cases we would observe in the year 2030 as specified in the EHE program in Chicago and Illinois. The predictive distribution for R_{2030} involves θ, which represents a generic combination of levers or pathway as an unknown prior; in this simulation we consider 2,304 combinations of these 6 levers; all but one combination involves more intensive leverage than our baseline present in 2015. The predictive distribution also involves the observed incidence for MSMs in Chicago for each of the years 2016, 2017, 2018, and 2019, which we sum and call $x_{2016-2019}$. We calculate the deviance D in terms of likelihood under a Poisson model between the sumulated count and the observed incidences $x_{2016-2019}$. We also define the random variable R_{2030} as the simulated numerical incidence of pathways in 2030. We formalize the relationships in a Bayesian framework. In particular, we place a uniform prior on the distribution of all 2,304 scenarios $(\theta i, i = 1, ..., 2, 304)$ of the 6 levers that we considered. Each scenario was replicated 44 times, i.e., pathways $\theta_{ij}, i = 1, ..., 2, 304 j = 1, ..., 44$. We calculated weights $W_{ij} = D_{ij} / \sum Di'j'$ for each replicated scenario. The posterior for θ given the data $x2016-2019$, is estimated by:

$$\widehat{\Pr}\left(\theta=\theta_i \mid \mathrm{X}_{2016-2019}\right)=\sum_{j=1}^{44} \mathrm{W}\left(\theta_{ij}\right) / \sum_{k=1}^{2304} \sum_{j=1}^{44} \mathrm{W}\left(\theta_{kj}\right) \tag{1}$$

The predictive distribution $f(r|x_{2016-2019})$ at 2030 depends on this posterior and is defined as the density:

$$f\left(r \mid x_{2016-2019}\right)=\sum_{k=1}^{2304} f\left(r \mid \theta_i, X_{2016-2019}=x_{2016-2019}\right) * \operatorname{Pr}\left(\theta=\theta_i \mid X_{2016-2019}=x_{2016-2019}\right) \tag{2}$$

This can be estimated using these weights in Equation (1). This distribution is equivalent to using what are called "pseudoclasses", which are random draws from the posterior distribution of an unobserved class (i.e., a pathway) given the data. Theoretical statistical work has demonstrated that this methods produces statistical summaries as well as the empirical predictive distribution are all asymptotically unbiased [1, 2].

References

[1] Wang CP, Hendricks Brown C, Bandeen-Roche K. Residual Diagnostics for Growth Mixture Models: Examining the Impact of a Preventive Intervention on Multiple Trajectories of Aggressive Behavior. Journal of the American Statistical Association. 2005 Sep;100(471):1054–1076. Available from: http://www.tandfonline.com/doi/abs/10.1198/016214505000000501.

[2] Petersen J, Bandeen-Roche K, Budtz-Jørgensen E, Groes Larsen K. Predicting Latent Class Scores for Subsequent Analysis. Psychometrika. 2012 Apr;77(2):244–262. Available from: http://link.springer.com/10.1007/s11336-012-9248-6.
