# Supplementary material for: Agent-based model projections for reducing HIV infection among MSM: Prevention and care pathways to end the HIV epidemic in Chicago, Illinois
Source: PLoS One. 2022 Oct 17;17(10):e0274288. doi: 10.1371/journal.pone.0274288 (PMC9576079; doi:10.1371/journal.pone.0274288)
Supplement: S3 Appendix — This appendix describes the details of the robustness check performed related to retention in care, and shows the experimental outcomes for two alternative ways of fitting the retention in care rates. (PDF) [file pone.0274288.s003.pdf]

Robustness Check for modeling outcomes

immediate

This supplementary information section provides details regrading the robustness of modeling outcomes. Specifically, it addresses model sensitivity to the assumption made in determining the rate of becoming unsuppressed while retained in care, which is part of the Treatment arm of the care system modeled in the Levers of HIV model. While there was no field data available for calculating the rate of becoming unsuppressed, a fitting process was used to determine the parameter value which minimizes the error in suppressed individuals among those in care and suppressed individuals among those diagnosed as living with HIV (People Living with HIV, or PLWH for short). While there is a trade-off between these criteria we had opted for a fit calculation that minimizes the following weighted mean squared error,

$$\text{wmse} = \left(\frac{m_d - a_d}{a_d}\right)^2 + \left(\frac{m_c - a_c}{a_c}\right)^2,$$

where m_d is the modeled ratio of suppressed individuals among those diagnosed HIV+. We know from CDPH surveillance data that in 2015 $a_d = 0.515$, the actual ratio of suppressed individuals among PLWH. Similarly, m_c is the modeled ratio of suppressed individuals among those in care, and from field data we know $a_c = 0.7961$, the actual ratio of suppressed individuals among those in care. While we consider our fitting process reasonable, as it optimizes fit with observed data, we want to explore if model results remain consistent when alternative methods of fitting are used. To this end we compare our results to those obtained by fitting either the suppressed individuals among those in care or suppressed individuals among PLWH (instead of compromising between both). The combination of these scenarios provide bounds to where the actual parameter would lie (while still producing somewhat realistic levels of suppression).

For each fitting criteria (Baseline, In-care (which focuses on fitting the rate of suppression among those in-care), and PLWH (which focuses on fitting the rate of suppression among PLWH) we determine the rate of becoming unsuppressed, and consequently compare the results of the main experiment by determining the optimal pathways towards attaining the 2030 goal of 90% reduction of new incidence cases. For each alternative fitting criteria the resulting decision tree is depicted below, yet for convenience these results are summarized in table 1.

Lever	Baseline	In-care	PLWH
ART linkage			
ART retention	Level = 3	Level = 3	Level = 3
Viral load (ART adherence)			
PrEP linkage	Level ≥ 2	Level ≥ 2	Level ≥ 3
PrEP retention	Level ≥ 2	Level ≥ 2	Level = 3
PrEP adherence	Level = 2	Level = 2	Level = 2
Prob. of success	58%	72%	72%

Table 1: comparison of optimal path towards attaining the 2030 EHE goal for the three alternative ways of fitting the rate of becoming unsuppressed

Our results show that there are slight differences across the fitting criteria use, both in terms of the rate of success, as well as the levels of perturbations required. While the baseline and In-care criteria differ only in the rate of success they predict (with baseline being the more conservative), the PLWH criteria imposes slightly stronger perturbations, requiring 1 level higher perturbations for both PrEP linkage as well as PrEP retention. Despite these small differences, we find that the general pathway towards attaining the 2030 EHE goals to remain remarkable stable, suggesting the modeled results are independent from the fitting criteria applied. As such, we deem or model results robust in lieu of the uncertainty in how the parameter for the rate of becoming unsuppressed is fitted.

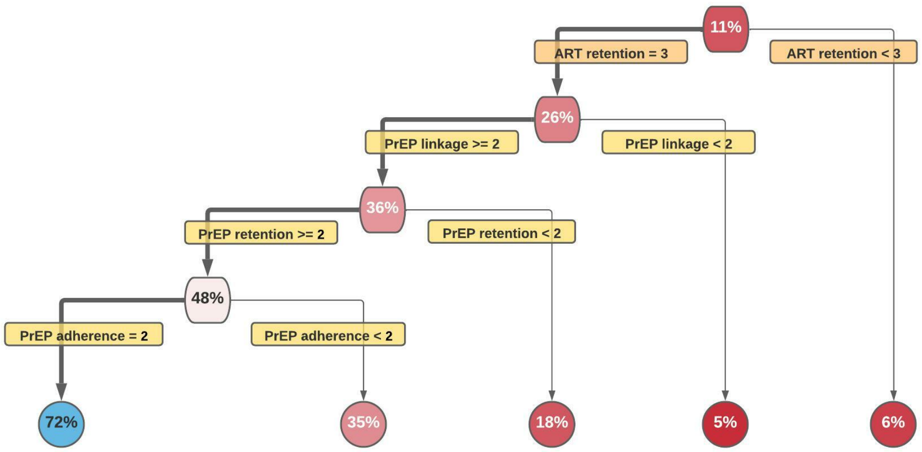

Figure 1: **Paths toward final EHE goal, 90% reduction of incidence by 2030, using the In-care fitting criteria .**

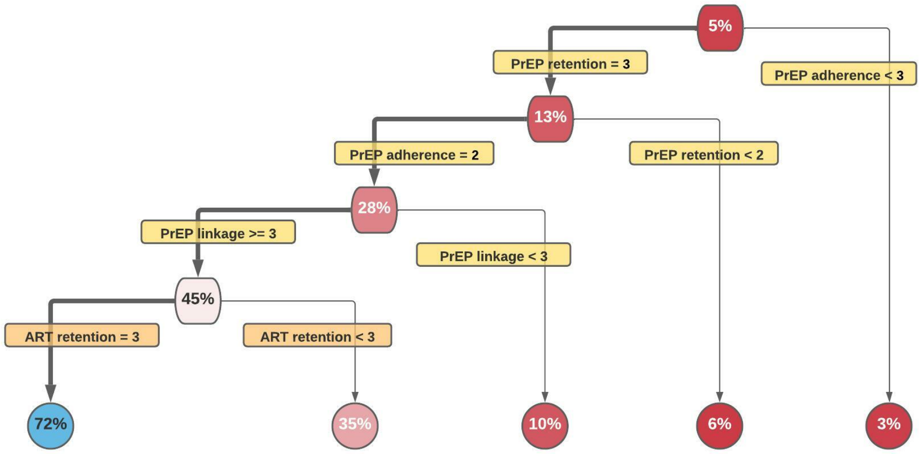

Figure 2: **Paths toward final EHE goal, 90% reduction of incidence by 2030, using the PLWH fitting criteria .**
