# Supplementary material for: Agent-based model projections for reducing HIV infection among MSM: Prevention and care pathways to end the HIV epidemic in Chicago, Illinois
Source: PLoS One. 2022 Oct 17;17(10):e0274288. doi: 10.1371/journal.pone.0274288 (PMC9576079; doi:10.1371/journal.pone.0274288)
Supplement: S4 Appendix — This appendix describes the details of the robustness check performed related to the rate of testing, and shows the experimental outcomes for two alternative rates of going in for HIV testing. (PDF) [file pone.0274288.s004.pdf]

Robustness Check for modeling outcomes - rates of testing

immediate

This supplementary information section provides details regrading the robustness of modeling outcomes. Specifically, it addresses model sensitivity to differences in the rate of testing among individuals. HIV testing is the first step towards potential initiation of preventive care (for those HIV negative) or treatment (for those HIV positive) and as such serves as a mechanism responsible for the inflow of individuals into the care system in our model. All individuals not in care will make a daily decision to go in for testing, the with the chance of actually going care being equal to the rate of testing. The 2016 National HIV Behavioral Surveillance data (NHBS) [1] states that in Chicago 77.1% of the MSM indicated they were tested in the last 12 months. Based on this data we parameterized our baseline model with an average rate of testing of once every 246 days, matching the proportion that gets tested annually. Note that in our implementation of the testing mechanism the rate of testing is chose to match the proportion of individuals being tested at least once a year, as this rate is well below once a year, this implies that a large proportion the population gets tested more than once each year. While we have no additional local details on the variance or heterogeneity in testing rates we applied this rate uniformly across the population. Given the limited data available to inform the local testing rates, we explore sensitivity of our model to changes in this rate to ensure the robustness of our findings. We introduce two alternative rates for testing; Alternative 1, in which on average 85% of MSM get tested annually, corresponding to an average testing rate of once every 192 days. And Alternative 2, in which on average 95% of MSM get tested annually, corresponding to a testing rate of once every 122 days. Similar to the baseline model we for each alternative conduct a full experiment, and produce a decision tree specifying the optimal path towards attaining the GTZ aim of 90% reduction in incidence by 2030.
The results of this analysis are summarized in Table 1 and show that there are slight differences across the different rates of testing. While alternative 1 shows a different order in splits and a increased rate of success in attaining the GTZ aim, it identifies an optimal pathway that is identical to our baseline. Alternative 2 shows again shows a different order in splits and a even further increased rate of success in attaining the GTZ aim, and differs in one key element; it shows that the increased rate of testing negates the

Lever	Baseline	Alternative 1	Alternative 2 path 1	Alternative 2 path 2
ART linkage				
ART retention	Level = 3	Level = 3		Level = 3
Viral load (ART adherence)				
PrEP linkage	Level $\geq$ 2	Level $\geq$ 2	Level $\geq$ 2	Level $\geq$ 2
PrEP retention	Level $\geq$ 2	Level $\geq$ 2	Level $\geq$ 2	Level $\geq$ 2
PrEP adherence	Level = 2	Level = 2	Level = 2	
Prob. of success	58%	74%	81%	70%

Table 1: Comparison of the paths towards attaining the 2030 EHE goal for the baseline and two alternative rates of testing.

need for retention in ART care. Based on these results, we concluded that increasing testing rates in isolation is insufficient to attain the GTZ goal, but does increase the likelihood of attaining this goal when combined with additional interventions. What is more, we find that extreme increases of testing rates (alternative 2) have the potential to negate either the need for interventions in retention to care (path 1) or the need for interventions in retention on PrEP (path 2). This is not a suprizing result when one considers that testing is synonymous with (re-)engagement in care. In other words, quickly reconnecting individuals to care, negates the need to retain them in care. Lastly, we consider our core PrEP pathway robust against increases in testing rate as it does remains stable in each of our testing scenarios.

References

[1] Centers for Disease Control and Prevention. HIV Infection Risk, Prevention, and Testing Behaviors among Men Who Have Sex With Men—National HIV Behavioral Surveillance, 20 U.S. Cities, 2014. 2016;(15):32. Available from: `https://www.cdc.gov/hiv/pdf/library/reports/surveillance/cdc-hiv-surveillance-special-report-number-15.pdf`.

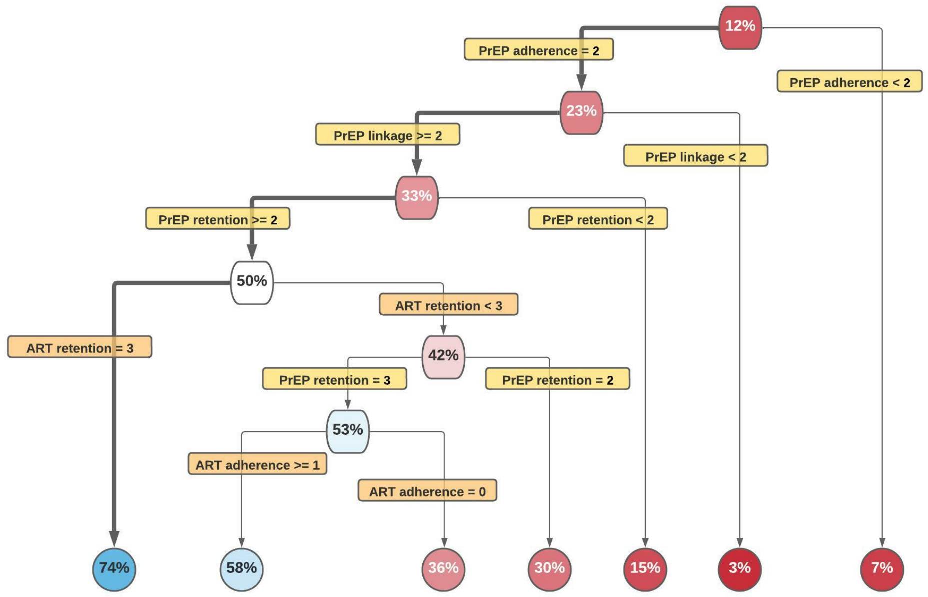

Figure 1: **Paths toward the EHE goal of 90% reduction of incidence by 2030, for alternative testing rate 1 (testing on average once every 192 days) .**

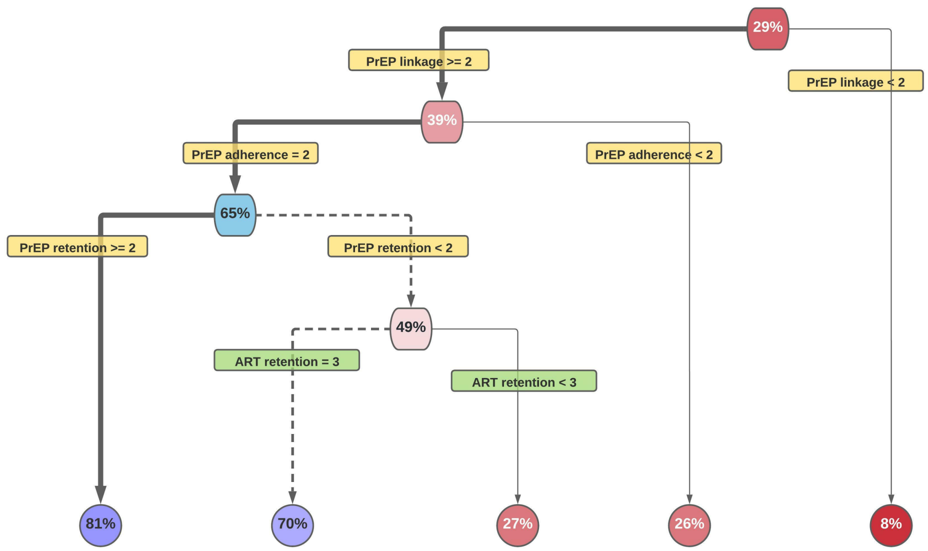

Figure 2: **Paths toward the EHE goal of 90% reduction of incidence by 2030, for alternative testing rate 2 (testing on average once every 122 days) .**
