# Supplementary material for: Agent-based model projections for reducing HIV infection among MSM: Prevention and care pathways to end the HIV epidemic in Chicago, Illinois
Source: PLoS One. 2022 Oct 17;17(10):e0274288. doi: 10.1371/journal.pone.0274288 (PMC9576079; doi:10.1371/journal.pone.0274288)
Supplement: Attachment — Submitted filename: comments of reviewer.docx [file pone.0274288.s005.docx]

This paper simulate the required changes in the local PrEP and ART cascade for meeting Chicago’s ’Getting to Zero’ goal of reducing new HIV diagnoses among MSM by 90% by 2030 using an agent-based model. They found that based on the current rate of decline (from 2016-2019) it is highly unlikely to achieve the 90% reduction target for 2030. In general, it can be said that valuable work has been done and deserves publication, however, the paper needs to be edited. The paper should be revised and edited by a professional editor to help improve the flow and its readability should be improved. I have some comments:

Introduction

1- In my opinion, the introduction section can be shorter and there are similar sentences in it, please summarize it.
2- Please be clear about the difference between your study and other studies that were conducted in Chicago and what the novelty of your study is.

Method

1- Please clarify that why you assume the proportion of MSM constant across age. Is it controllable in the ABM.
2- Please clarify that why you consider only 10 percent of LHM population.
3- Please clarify that why you not consider HIV testing as intervention in the model. Increasing testing frequency has the largest impact on reduction in HIV infections

Result

1- Please correct the symbols (e.g. <=) in the results text.

Discussion

1- It seems that the titles of the discussion and conclusion sections have been written in place of each other. Limitations should be in the discussion section.
2- In the discussion section, please compare your results with other studies that have used deterministic mathematical model and ABM.

Conclusion

1- The conclusion should be more concise. Please summarize it.
