# Supplementary material for: Agent-based model projections for reducing HIV infection among MSM: Prevention and care pathways to end the HIV epidemic in Chicago, Illinois
Source: PLoS One. 2022 Oct 17;17(10):e0274288. doi: 10.1371/journal.pone.0274288 (PMC9576079; doi:10.1371/journal.pone.0274288)
Supplement: Attachment — Submitted filename: Vermeer - Revision 1 - response to reviewer comments.docx [file pone.0274288.s006.docx]

Dear reviewers,

We thank you for your time in reviewing our manuscript and your constructive feedback. Based on the comments provided we have identified two main areas for improvement for this manuscript; improving the flow and structure of the paper, and providing further details on model assumptions. In this revised manuscript we have addressed these accordingly. First, we altered the flow of the paper by shortening the introduction and conclusion sections and expanded the discussion section. Second, we have provided additional clarifications throughout the paper and added a section on the impact of testing. Third, we had the manuscript edited by professional editor to improve readability. In addition to these larger changes, we present responses to each of the reviewer’s comments below.

We hope our modifications are satisfactory and look forwards to the next steps in the review process.

Kind regards,

Wouter Vermeer

**Reviewer #1:**

The paper highlights a critical issue profoundly and comprehensively. However, I have the following recommendations:

**1. it is too long, they may shorten the introduction and even the other parts by dropping or summarizing less relevant sentences**

As part of our process of adjusting the flow of the paper we have shortened the introduction section and removed less relevant sentences.

**2. the title of graphs and tables and the headers are not fully explained. For example, what is the scale of incidence in table 3 and figure 2?**

We have edited Table 3, Figure 2, Figure 6, Figure 7, and Figure 8 to clarify that “annual incidence” on the y-axis refers to the annual count of incident cases.

**3. the discussion is too short, and most of the main parts of the discussion were relocated in the conclusion. They have to organize their discussion accordingly. For example, limitations and the comparison between their results with others' findings are the main parts of the discussion**

As part of our restructuration of the paper we have reorganized the discussion and conclusion sections. The revised discussion section compares our results to previous work, provides guidelines for interpretation, and describes limitations of our model. The conclusion section provides a short, condensed summary of our finding and implementations.

**4. limitations and their impact on the final results are significant in such a deep and extended modeling. They have to explain this part in more detail and even paste a table to organize the main limitations and their possible impact on the final results. For instance, they only explored the MSM subpopulation, while it has a strong link with the other most at-risk sub-populations, which may change the results of this study if the protection and the prevention measures change simultaneously.**

We have edited the limitations of our model and our methods and added implications on how results could change. We attempted to summarize these in a table but found the flow of this section was easier to read in text form. We have reorganized need for clarification of the limitations and put additional emphasis on the implications and generalizability stemming from the limitations.

**5. If they have access to the field data in more recent years to compare their projections with exact numbers to show the accuracy of their predictions.**

The most recent field data currently available covers the period up to 2020, these data are generally provided in annual reports; COVID delayed the reporting. Based on the 2020 data we show that modeled baseline levels of incidence based on 2015 behaviors trail behind the observed level for the period 2016-2019 (Figure 6)..

**Reviewer #2:** This paper simulates the required changes in the local PrEP and ART cascade for meeting Chicago’s ’Getting to Zero’ goal of reducing new HIV diagnoses among MSM by 90% by 2030 using an agent-based model. They found that based on the current rate of decline (from 2016-2019) it is highly unlikely to achieve the 90% reduction target for 2030. In general, it can be said that valuable work has been done and deserves publication, however, the paper needs to be edited. The paper should be revised and edited by a professional editor to help improve the flow and its readability should be improved. I have some comments:

**Introduction
1- In my opinion, the introduction section can be shorter and there are similar sentences in it, please summarize it.**

We have reduced the introduction and removed similar sentences in the introduction section.

**2- Please be clear about the difference between your study and other studies that were conducted in Chicago and what the novelty of your study is.**

We have added a section (L132-144) that specifies how our model differs from existing modeling work, both within Chicago and more broadly, and how it is novel in this domain.

**Method**

**1- Please clarify why you assume the proportion of MSM is constant across age. Is it controllable in the ABM.**

We assumed constancy across age and race/ethnicity because no additional data were available other than an overall value. We have edited lines 180-181 as follows: “

*As detailed local data is missing, our model assumes that the proportion of MSM is constant across race/ethnicity and age*”

**2- Please clarify that why you consider only 10 percent of LHM population.**

Our choice was solely based on computational resources required for running our experiments.

While ABM conceptually allows for 1:1 modeling of populations, doing so comes at substantial cost of computational resources. In our 1:10 model the complex behavior exhibited by each agent, and specifically among interacting agents, required some scaling to be implemented. For example, in each time-step all agents seeking partners requires calculation of a relative match for all others seeking partners, which becomes resource intensive as the number of agents increase. Furthermore, while a single 1:10 run is relatively fast (roughly 30 mins) a complete experiment of 2304 scenarios with 44 repetitions (~100k runs) takes roughly 50k computational hours, or over 4 days on a 100-core cluster. Running a full-scale 1:1 model experiment would require more than a month which is simply not a feasible resource allocation.

We early on explored the relative impact on system behaviors of scaling. Scaling primarily impacts the partnership selection and consequently interaction networks formed (which is also why computational cost go up closer to exponential rather than linearly). In our explorations we found no significant impact on the systemic dynamics or incidence rates as a result of the reduced scales.

We included a simple addition to Line 210 which now reads:

*“To reduce computational requirements of our experiments the LHM samples 10% of this population and contains 6500 individuals, describing the city on a 1:10 scale while maintaining the race/ethnicity, age, and location data from the Chicago census, and HIV prevalence distributions by these demographic conditions from the CDPH surveillance data.*”

**3- Please clarify why you do not consider HIV testing as intervention in the model. Increasing testing frequency has the largest impact on reduction in HIV infections**

We are aware of the potential impact of testing/screening/diagnosis, it is the conduit into the prevention and care systems (e.g., increasing testing would increase linkage) and thus an important component to what we have already examined. We did run additional simulations specifically varying testing rates, but because the very limited information on testing, we retained our 6 levers for the paper and summarized findings of testing in a new supplemental section.

We base our rate testing in our model on the observation made in the 2016 National HIV Behavioral Surveillance data (NHBS) that states that among MSM in Chicago 77.1% had been tested in the last 12 month. We do not know how this rate varies by race/ethnicity or age, nor do we know the how often individuals would get more than one test per year.

We agree that the manuscript would benefit from highlighting the potential of testing/screening in changing the system dynamics and have added language to the main text where we recognize it as a source of uncertainty and included the findings of a sensitivity analysis exploring the impact of changes in the testing rate (lines 388-392, and 572-583). These findings are presented in detail a 4^th^ supplementary information section.

The sensitivity analysis reveals that while testing increases alone will not achieve GTZ goals, improvements in the rates of testing are likely to result in a higher likelihood of attaining set goals when combined with interventions in the prevention and treatment cascade. What is more we find that extreme increases in testing rate, might negate the need to either retention in treatment, or retention in care, which is not a surprising finding when one considers that testing is the mechanism responsible also to re-engagement in care. Effectively stating that keeping people in treatment/prevention is less critical when they would re-enter set system (by getting tested) shortly after discontinuation.

**Result
1- Please correct the symbols (e.g. <=) in the results text.**

Thanks for identifying this, corrections have been made.

**Discussion
1- It seems that the titles of the discussion and conclusion sections have been written in place of each other. Limitations should be in the discussion section.
2- In the discussion section, please compare your results with other studies that have used deterministic mathematical model and ABM.**

We have revised the discussion accordingly.

**Conclusion
1- The conclusion should be more concise. Please summarize it.**

As mentioned previously in this response, as part of our restructuration of the paper we have reorganized the discussion and conclusion sections. In the current version the discussion holds a comparison of our results to previous work, guidelines for interpretation, and limitation of our model. Whereas the conclusion provides a short, condensed summary of our work.
